# Correcting for link loss in causal network inference caused by regulator interference

**DOI:** 10.1093/bioinformatics/btu388

**Published:** 2014-06-19

**Authors:** Ying Wang, Christopher A. Penfold, David A. Hodgson, Miriam L. Gifford, Nigel J. Burroughs

**Affiliations:** ^1^Warwick Systems Biology Centre and ^2^School of Life Sciences, University of Warwick, Coventry CV4 7AL, UK

## Abstract

**Motivation:** There are a number of algorithms to infer causal regulatory networks from time series (gene expression) data. Here we analyse the phenomena of regulator interference, where regulators with similar dynamics mutually suppress both the probability of regulating a target and the associated link strength; for instance, interference between two identical strong regulators reduces link probabilities by ∼50%.

**Results:** We construct a robust method to define an interference-corrected causal network based on an analysis of the conditional link probabilities that recovers links lost through interference. On a large real network (*Streptomyces coelicolor*, phosphate depletion), we demonstrate that significant interference can occur between regulators with a correlation as low as 0.865, losing an estimated 34% of links by interference. However, levels of interference cannot be predicted from the correlation between regulators alone and are data specific. Validating against known networks, we show that high numbers of functional links are lost by regulator interference. Performance against other methods on DREAM4 data is excellent.

**Availability and implementation**: The method is implemented in R and is publicly available as the NIACS package at http://www2.warwick.ac.uk/fac/sci/systemsbiology/research/software.

**Contact:** N.J.Burroughs@warwick.ac.uk

**Supplementary information:** Supplementary materials are available at *Bioinformatics* online.

## 1 INTRODUCTION

The falling costs of global gene expression (transcriptome) measurement by microarrays, and more recently next-generation sequencing, has spurred the development of network inference techniques (Bansal *et al.*, 2007; De Smet and Marchal, 2010; Emmert-Streib *et al.*, 2012; Hache *et al.*, 2009; Maetschke *et al.*, 2013; Margolin and Califano *et al.*, 2007; Markowetz and Spang, 2007; Olsen *et al.*, 2009; Penfold and Wild, 2011; Werhli *et al.*, 2006). Of particular interest, given their direct bearing on mechanisms of regulation and information flow through the cell, is the identification of causal regulatory signals, the associated directed (causal) networks and regulatory pathways. Time series experiments potentially capture these signals, but because of low temporal resolution of transcriptome data, only simple models can be used for analysis. The most basic is a linear auto-regression model (Lebre, 2009; Morrissey *et al.*, 2010; Opgen-Rhein and Strimmer, 2007),

(1) $$X_{i}^{t+1}=\mu_{i}+\sum_{j=1}^{G}B_{ij}X_{j}^{t}+\varepsilon_{i}^{t},$$

where $X_{i}^{t}$ is the (log) expression level of gene *i* (i=1,…,G) at time *t* (t=1,…,T), *B_ij_* is the connectivity strength, $\mu_{i}$ the additive constant and $\varepsilon_{i}^{t}$ Gaussian noise $N(0,\lambda_{i}^{-1})$, (precision $\lambda_{i}$).

Despite the simplicity of these models, inference is computationally intensive given the high number of regressors; expression data on 1000–10 000s of genes is typical depending on the experimental conditions and organism, and potentially greater if gene models (RNA splicing/transcripts) are distinguished. A factor that considerably aids identification of regulatory links in these systems is the fact that biological networks are sparse, i.e. the connectivity matrix $B=(B_{ij})$ is sparse with the average number of regulators per gene being much smaller than the number of genes. Part of this sparsity results from the fact that only a subset of genes (more specifically, their associated proteins) can be regulators; the set of potential regulators can thus be restricted a priori to those identified from bioinformatic/literature considerations as potential regulators, thereby reducing computation considerably.

Sparse network models (Morrissey *et al.*, 2010, 2011) use Gibbs variable selection methods to determine which elements of the matrix *B* are present in the regression; specifically, the prior on *B_ij_* allows it to be zero with finite probability. The indicator $\gamma_{ij}\in \{0,1\}$ of a link j→i determines if *B_ij_* is non-zero (when $\gamma_{ij}=1$), while *B_ij_* = 0 if $\gamma_{ij}=0$. The indicator γ is modelled as a Bernoulli distribution with a prior probability ρ of being non-zero, $\pi (\gamma_{ij}\;|\;\rho )∼Ber(\gamma_{ij}\;|\;\rho )$, while ρ has a Beta prior π(ρ)∼Be(ρ|0.5,0.5) (Morrissey *et al.*, 2010). Let *D* denote the time series gene expression data $\left\{X_{i}^{t}\right\}$ and θ the model parameters $\left\{(\mu_{i}),(\lambda_{i}),(\gamma_{ij}),(B_{ij})\right\}$. The likelihood is then given by

(2) $$L(\theta ;D)=\prod_{i=1}^{G}\prod_{t=1}^{T-1}N(X_{i}^{t+1}\;|\;\mu_{i}+\sum_{j=1}^{G}\gamma_{ij}B_{ij}X_{j}^{t},\lambda_{i}^{-1}).$$

The associated posterior can then be sampled using the bioconductor package GRENITS (http://www.bioconductor.org/packages/2.12/bioc/html/GRENITS.html).

In network models, interference has a direct bearing on the posterior probability of links being present. Specifically in these sparse network models, $\pi (\gamma_{ij}=1\;\;|\;\;D)$ is reduced if there is another regulator *k* with similar dynamics to *j*. In the case of an exact identity between the dynamics of *j* and *k*, there are three identical regression models: $\gamma_{ij}=1,\gamma_{ik}=0$; $\gamma_{ij}=0,\gamma_{ik}=1$ and $\gamma_{ij}=1,\gamma_{ik}=1$, and thus the probability of any one, and therefore of $\pi (\gamma_{ij}=1\;|\;D)$ is reduced. The relative weighting of these three states is determined by the prior link probability ρ, which is low in sparse networks, thereby downweighting the double link case; therefore, only the two states $\gamma_{ij}=0,\gamma_{ik}=1$ and $\gamma_{ij}=1,\gamma_{ik}=0$ need to be considered, effectively halving $\pi (\gamma_{ij}=1\;|\;D)$ relative to the case when *k* is excluded from the network (proof in Section 2.1.1). A key problem is gauging when regulators might interfere, specifically how interference decreases with a diminishing correlation between these regulators and thus deciding how to select which regulators to use in the regression. Failing to deal with this appropriately means that key regulators might be missed because they are part of a correlated set of regulators, and their link probabilities fall below threshold through mutual interference. We developed a framework for solving this problem based on the analysis of conditional posterior link probabilities that identifies the interfering sets of regulators. This allowed us to define an interference-corrected causal network and, further, the relative weights of the interfering regulators reflecting their likely contribution to the control of a given gene.

The article is organized as follows. In Section 2, we analyse the impact of identical regulators on network links, demonstrating that the link probabilities of identical strong regulators are essentially additive. We give a numerical demonstration of the theory on an augmented experimental dataset, doubling up a key regulator. In Section 3, we develop a framework to correct for interference in network construction, a post-processing step that clusters regulators and calculates regulator interference within clusters for each target. In Section 4, we illustrate our method on three experimental datasets that give rise to networks with distinct architectures and demonstrate that interference is data specific, there being no simple relationship between interference and regulator correlation. Further, we provide evidence that the recovered links are functional. In Section 5, we discuss the impact of these issues and the generality to other inference/fitting methods.

## 2 THE REGULATOR INTERFERENCE PROBLEM

Causal network determination relies on causal signals leaving a signature in the gene expression dynamics, essentially a correlation between $X^{t+1}$ and $X^{t}$. However, if two (or more) regulators have similar dynamics, $X_{j}^{t}\approx X_{k}^{t}$ for all *t*, then the identity of the regulator is unclear, the true regulator being either *j*, *k* or both. This correlation in the data gives rise to an approximate symmetry for the likelihood, $L(\theta ;X^{t})\approx L(\theta ;\Phi_{j,k}X^{t})$, where $\Phi_{j,k}$ is the exchange transformation j↔k. Lifting the symmetry to the space of model parameters gives rise to an approximate exchange symmetry on the parameters θ, $L(\theta ;X^{t})\approx L(\Phi_{j,k}^{†}\theta ;X^{t})$. In particular, identical data $X_{j}=X_{k}$ means that the likelihood has an invariance symmetry, and *B_ij_* and *B_ik_* are unidentifiable in the linear network model (1). The lifted symmetry in this case reads $\Phi_{j,k}^{†}B_{\cdot j}=B_{\cdot k}$, $\Phi_{j,k}^{†}B_{j\cdot }=B_{k\cdot }$, etc. In this section, we develop a framework to analyse and detect interference between regulators and demonstrate the impact of an exact symmetry (two identical regulators) on real data.

### 2.1 Two identical regulators

#### 2.1.1 Theoretical analysis of regulator interference

Consider an exact data symmetry $\Phi_{j,k}X^{t}=X^{t}$, i.e. *X_j_*, *X_k_* are identical. The likelihood is then invariant under the lifted symmetry $\Phi_{j,k}^{†}$. The prior is also likely to satisfy this symmetry, i.e. the prior on *B_ij_* is likely identical to that of *B_ik_*. Thus, the posterior link probabilities satisfy this symmetry, $\pi (\gamma_{ij}\;|\;D)=\pi (\gamma_{ik}\;|\;D)$ and similarly all joint conditionals. However, the aspect of the symmetry we are interested in is whether the following two models predict different networks.

M1 Only regulator *j* is considered in the set of regulators, i.e. regulator *k* is removed from the network.

M2 Both regulators *j*, *k* are present in the network.

These are nested networks because M1 is M2 under the constraint $\gamma_{ik}=0$, i.e. the conditional posterior probability $\pi (\gamma_{ij}=1\;|\;D,\gamma_{ik}=0)$ is the link probability on M1, whereas that of M2 is the posterior probability $\pi (\gamma_{ij}=1\;|\;D)$, (no constraints), both of which can be computed from the joint distribution $\pi (\gamma_{ij},\gamma_{ik}\;|\;D)$.

We proceed to determine an expression relating $\pi (\gamma_{ij}=1\;|\;D,\gamma_{ik}=0)$ and $\pi (\gamma_{ij}=1\;|\;D)$. Throughout we consider ρ as fixed; in practice, it has low posterior variance in the full network analysis and so fixing ρ equal to the posterior mean in the following is a good approximation. Firstly, we have

$$\pi (\gamma_{ij}=1,\gamma_{ik}=1\;|\;D)=\frac{\rho^{2}}{\pi (D)}\int dB_{ij}dB_{ik}\pi (B_{ij})\pi (B_{ik})$$

$$\;\int d\tilde{\theta }\pi (\tilde{\theta })L(\tilde{\theta },B_{ij},B_{ik},\gamma_{ij}=1,\gamma_{ik}=1;D)$$

using Bayes formula and assuming independent priors. Here ρ is the (fixed) link prior probability and $\tilde{\theta }=\theta \setminus \{B_{ij},B_{ik},\gamma_{ij},\gamma_{ik}\}$.

The data symmetry $\Phi_{jk}$ between *j*, *k* implies that $L(\tilde{\theta },B_{ij}=a,$ $B_{ik}=b,\gamma_{ij}=1,\gamma_{ik}=1;D)=L(\tilde{\theta },B_{ij}=a+b,\gamma_{ij}=1,\gamma_{ik}=0;D)$. With a Gaussian prior on $B_{ij}∼N(0,\sigma^{2})$ (variance $\sigma^{2}$), we note that for an arbitrary function *f*,

$$\int dudve^{-\frac{\left(u^{2}+v^{2}\right)}{2\sigma^{2}}}f(u+v)\equiv \int \frac{1}{2}dwdze^{-\frac{\left(w^{2}+z^{2}\right)}{4\sigma^{2}}}f(w)$$

$$\;=\sqrt{\pi }\sigma \int dwe^{-\frac{w^{2}}{4\sigma^{2}}}f(w)$$

and thus deduce that,

$$\pi (\gamma_{ij}=1,\gamma_{ik}=1\;|\;D,\sigma^{2})=\frac{\rho }{1-\rho }\pi (\gamma_{ij}=1,\gamma_{ik}=0\;|\;D,2\sigma^{2})$$

where we make explicit note of the prior variance $\sigma^{2}$ on $B_{ij},B_{ik}$ because the dimension reduction above has doubled the prior variance. This implies that the symmetry $\Phi_{jk}$ results in a direct relationship between the regulator link probability j→i when one (Model M1) or both links (M2) are present up to changing the Gaussian prior variance of *B_ij_* between Models M1 and M2; the other strength coefficients Bis,s≠j retain the original prior. Assuming a sufficiently weak prior, we thus obtain an approximate equality $\pi (\gamma_{ij}=1,\gamma_{ik}=1\;|\;D)\approx \frac{\rho }{1-\rho }\pi (\gamma_{ij}=1,\gamma_{ik}=0\;|\;D)$.

Therefore, we deduce that

$$(1-\rho )\pi (\gamma_{ij}=1\;|\;D)\approx \pi (\gamma_{ij}=1,\gamma_{ik}=0\;|\;D),$$

which gives

(3) $$\pi (\gamma_{ij}=1\;|\;D,\gamma_{ik}=0)\approx \frac{\left(1-\rho )\pi (\gamma_{ij}=1\;|\;D\right)}{1-\pi (\gamma_{ij}=1\;|\;D)},$$

or

(4) $$\pi (\gamma_{ij}=1\;|\;D)\approx \frac{\pi (\gamma_{ij}=1\;|\;D,\gamma_{ik}=0)}{1-\rho +\pi (\gamma_{ij}=1\;|\;D,\gamma_{ik}=0)}.$$

Hence, including an identical regulator in a network reconstruction reduces the link probability j→i by a factor of $1-\rho +\pi (\gamma_{ij}=1\;|\;D,\gamma_{ik}=0)$, which can be close to 2 for a strong link ($\pi (\gamma_{ij}=1\;|\;D,\gamma_{ik}=0)\approx 1$) in a sparse network (ρ low). For weaker connections, the reduction factor is smaller but the relative change proportionally greater, growing as $1+[(1-\rho )/\pi (\gamma_{ij}=1\;|\;D,\gamma_{ik}=0)]$.

#### 2.1.2 Demonstration of regulator interference on experimental data

We use time series data for *Streptomyces coelicolor* under phosphate depletion (Nieselt *et al.*, 2010), expression data consisting of 19 time points with 1 h sampling (see Section 1.1 of Supplementary Material). In bacteria, the PhoP two-component system (PhoURP, SCO4228-30 in *S.coelicolor*) is the primary response pathway during phosphate depletion (Rodriguez-Garcia *et al.*, 2007); transcription of *phoP* (SCO4230) dramatically increasing within 1 h of depletion (Nieselt *et al.*, 2010), a signalling cascade that gives rise to antibiotic synthesis within 20 h (Wentzel *et al.*, 2012) through activation of the *red* (undecylprodigiosin, RED) and *act* (actinorhodin, ACT) gene clusters. For demonstration purposes, we selected a set of 120 differentially expressed (DE) genes comprising 65 predefined DE regulators (removing *phoU/R* that have a high correlation with *phoP*; the interference of these three regulators is analysed in Section 4.1) together with a selection of *phoP*-dependent genes and *phoP*-independent genes. We denote this dataset $D^{1\times phoP}$ to reflect that it has one copy of the *phoP* gene. We constructed an augmented dataset by including one additional artificial regulator, which we called SCO0000, with expression data that is an exact copy of *phoP*. We called this dataset $D^{2\times phoP}$ because it has two regulators (SCO4230, SCO0000) with identical gene expression.

**Fig. 1.**
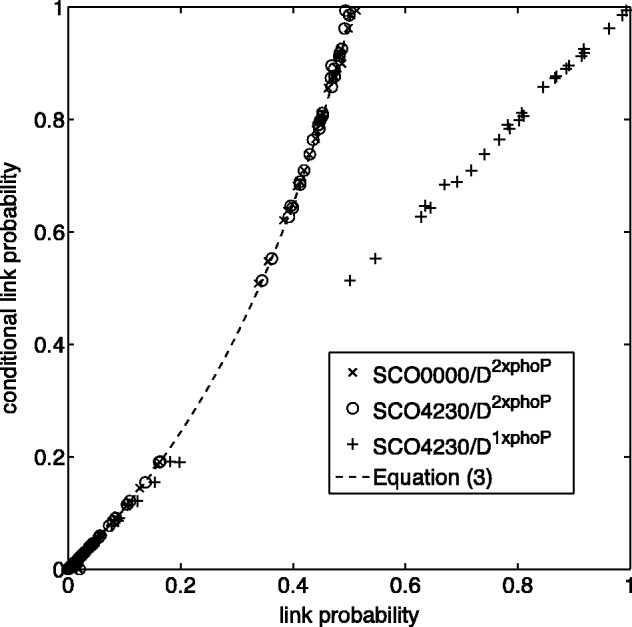
Link probability suppression in the presence of an identical regulator. For each target, the conditional link probabilities of SCO4230 (°) and SCO0000 (×) (conditioned on the other regulator being off) are plotted against their posterior link probabilities for the network inferred on the dataset $D^{2\times phoP}$ comprising 121 genes, including the regulator SCO4230 and its artificial copy SCO0000. The conditional link probability is computed from the MCMC samples (18 000 samples) by conditioning on the respective duplicate being off. The theoretical relationship, Equation (3) with ρ=0.026 (posterior mean), is shown (dashed line). The conditional link probability of SCO4230 on $D^{2\times phoP}$ is also plotted against the link probability of SCO4230 inferred on $D^{1\times phoP}$ (+), the corresponding dataset that lacks the artificial regulator SCO0000

The bioconductor package GRENITS was used for inference of the gene regulatory network on $D^{1\times phoP}$, and $D^{2\times phoP}$ (see Section 2 of Supplementary Material). GRENITS uses a Markov chain Monte Carlo (MCMC) algorithm to implement Bayesian inference on the sparse network model (1), giving samples of the network from which link probabilities can be calculated. For the duplicated dataset $D^{2\times phoP}$, we calculated the conditional link probabilities for SCO4230 and SCO0000 from the MCMC samples, i.e. the probability of SCO4230 being on while SCO0000 is off, and *vice versa*. As is shown in Figure 1, the link probabilities for SCO4230 decreased dramatically when its duplicate SCO0000 was included relative to the conditional probability when the duplicate was switched off. This demonstrates the presence of regulator interference in causal networks under an exact data symmetry. Our analysis also demonstrates that the conditional probability allows the link probability to be accurately reconstructed compared with network inference on a reduced set of regulators, i.e. removal of the artificial (duplicated) gene SCO0000 in this case. Specifically, the conditional $\pi (\gamma_{iSCO4230}\;|\;D^{2\times phoP},\gamma_{iSCO0000}=0)$ is identical to $\pi (\gamma_{iSCO4230}\;|\;D^{1\times phoP})$ for each target *i*, lying down the diagonal of Figure 1. Therefore, up to the proviso of sufficient samples to compute the conditional, all analysis can be performed from one run of the MCMC sampler through a post-processing step. Further, as the conditional probabilities of SCO4230 and SCO0000 agree (conditioned on each other), both lying on the theoretical curve (3) (Fig. 1) the GRENITS sampler mixes well under this data duplication, i.e. an identifiability symmetry between regulators does not affect mixing.

### 2.2 Multiple identical regulators

For *n* identical regulators *S_i_*, $\left|S_{i}\right|=n$, of the target *i*, the regulator interference analysis of Section 2.1.1 generalizes to give the approximate relationship (see Section 4.1 of Supplementary Material)

$$\pi (\gamma_{ij}=1\;|\;D,\gamma_{ik}=0,\forall k\in S_{i}\setminus \{j\})$$

$$\approx \frac{{\left(1-\rho \right)}^{n-1}\pi (\gamma_{ij}=1\;|\;D)}{1-\frac{1-{\left(1-\rho \right)}^{n-1}}{\rho }\pi (\gamma_{ij}=1\;|\;D)}.$$

For strong links in a sparse network (ρ small), we get the approximate relation

$$\pi (\gamma_{ij}=1\;|\;D)\approx \frac{\pi (\gamma_{ij}=1\;|\;D,\gamma_{ik}=0,\forall k\in S_{i}\setminus \{j\})}{n}$$

Thus, interference by identical regulators in sparse networks reduces link probabilities by the number of identical genes, the network topology running randomly over the single regulator networks because states with multiple links are severely down weighted by ρ. The regulators thus share equally the causal signal, and the true link probability of the causal dependence is (approximately) given by the sum over the link probabilities of these interfering regulators.

## 3 DETECTING NETWORK LINK INTERFERENCE

A continuity argument to the identical regulator case above, Section 2.1.1, suggests that we should expect interference to occur between highly correlated regulators. However, the dependence of interference (link suppression) on the degree of correlation between the regulators, the expression levels of the regulators and their targets, is unknown. What is clear is that correlated genes can interfere with each other, potentially causing a significant reduction in the posterior link probabilities of dynamically similar regulators by as much as a factor of *n* for *n* highly correlated regulators. Therefore, causal networks defined by application of a simple threshold criterion, $\pi (\gamma_{ij}=1\;|\;D)>\phi$, are potentially erroneous because interfering regulators may be lost since their posterior link probabilities fall below the threshold *ϕ*. Here we establish a method to construct a network corrected for interference.

Our method is as follows. Given expression (time series) data *D* (restricted to DE genes), and a subset *R* of potential regulators based on, for example, functional annotation, define the interference-corrected network (ICN) N(ω,ϕ) with correlation and link thresholds ω, *ϕ* respectively, by

1. Cluster the regulators *R* into correlated sets $\;|\;C_{jk}\;|\;\geq \omega$, using the correlation coefficient (or other similarity index) *C_jk_*, defining clusters $CR^{s}(\omega ),s=1,2...$, possibly singletons. We determine levels of interference between regulators within each cluster, ignoring interference between regulators outside these sets. Singleton clusters are regulators where interference from all other regulators is ignored.
2. Infer the network posterior distribution and the conditional posterior link distributions for regulators in the clusters (here we use MCMC samples from GRENITS). Specifically, compute for each $j\in CR^{s}(\omega )$ the conditionals $\pi (\gamma_{ij}=1\;|\;D,\gamma_{ik}=0,\forall k\in CR^{s}(\omega )\setminus \{j\})$ for each target *i*.
3. For a regulator $j\in CR^{s}(\omega )$ and a target *i*, a link j→i in the network N(ω,ϕ) exists if either of the following two conditions holds
  $$\alpha \text{-link}:\pi (\gamma_{ij}=1\;|\;D,R)\geq \phi .$$
  $$\beta \text{-link}:\pi (\gamma_{ij}=1\;|\;D,R)<\phi \;\text{and}$$
  $$\;\pi (\gamma_{ij}=1\;|\;D,R,\gamma_{ik}=0\forall k\in CR^{s}(\omega )\setminus \{j\})\geq \phi .$$

For singletons, only the α-link condition is relevant. A further numerical restriction is also applied to β-links; the sample size for calculating the conditional probability should be ≥100 in order that a 90% confidence interval can be computed; on smaller sample sizes, accuracy of this estimate is poor. Small sample sizes can occur if there is a very strong link in the cluster, then conditioning on that link to be off gives a small number of samples. Acquiring a larger number of posterior samples (running the sampler for longer) relaxes this limitation.

The α-links in N(ω,ϕ) are the original links in N(1,ϕ) (uncorrected network), i.e. the ICN comprises the original network augmented with recovered β-links. We define the link probability for each link in the ICN as the maximum of its posterior link probability and the links' conditional probability based on the regulator’s cluster at threshold ω (if applicable).

The number of β-links in the ICN increases on average as the correlation threshold ω decreases for a fixed link probability threshold *ϕ*. However, as the correlation threshold decreases, interference amongst clustered regulators becomes weaker as regulators with decreased levels of mutual interference are clustered together. Therefore, to find a correlation threshold where the partitioning of regulators into mutually interfering sets is optimized, we use a scoring method that quantifies levels of interference. We propose a score where a β-link has a weight inversely proportional to the number of regulators in its cluster $CR^{s}(\omega )$, giving the β-link network score

(5) $$S(\omega ,\phi )=\sum_{s}\sum_{j\in CR^{s}}\sum_{i}\frac{I(j\to i)}{\;|\;CR^{s}(\omega )\;|\;}$$

where I(j→i) is 1 if the β-link is present, otherwise 0. Then strong interference among highly correlated regulators increase the score, while weaker regulators (where only a proportion of the regulators in the cluster have a conditional probability above threshold to a given target) decrease the score. For instance, forming a new cluster (ω decreasing) comprising two β-links adds 1 to the score. But adding a third regulator to the cluster that does not regulate that target (conditional below threshold) reduces the contribution to the score to 2/3. Typically β-links are stable in that those present at ω are a subset of β-links for all ω′<ω. We maximize the network score S(ω,ϕ) with respect to ω, defining the optimal correlation threshold $\omega^{*}(\phi )$ and associated optimal ICN. In cases where there is a tie, we use the highest correlation that maximizes the β-link network score because further clustering gives no evidence of an increase in interference.

The link probability threshold *ϕ* is chosen based on the confidence required for assigning links (Morrissey *et al.*, 2010, 2011); in practice, it is lower than desirable because posterior link probabilities are typically low (see Supplementary Fig. S4). We note that the value ϕ=1/(2−ρ) has a special significance as regards interference analysis (the prior link probability parameter ρ is fixed at its posterior mean), as it is the highest value of the conditional link probability for two identical regulators, see Equation (4) with $\pi (\gamma_{ij}=1\;|\;D,\gamma_{ik}=0)=1$. Thus, when ϕ<1/(2−ρ), some of the α-links are interfering with each other, as shown in Figure 1. Hence, to correctly assess interference, the link threshold should be greater than 1/(2−ρ). In Section 4, we consider the effect of *ϕ* on interference and its impact on $\omega^{*}(\phi )$ on real datasets.

Finally, we note that different clustering methods are likely to give slightly different results for the ICN, but identification of interfering regulators is likely similar.

## 4 DEMONSTRATIONS ON REAL DATA NETWORKS

In this section, we analyse interference on three experimental examples. Firstly, the full expression dataset for *S.coelicolor* under phosphate depletion (Nieselt *et al.*, 2010), a second experimental dataset for *S.coelicolor* under glutamate depletion (Waldvogel *et al.*, 2011) and expression data for the *Arabidopsis* circadian clock (Windram *et al.*, 2012) (see Section 1 of Supplementary Material). In each case, we do not subselect regulators based on their (dynamic) similarity but construct the causal network with all potential regulators present. We demonstrate in two of the networks that a high proportion of the recovered links are functional and quantify network accuracy.

### 4.1 Example 1: phosphate depletion causal network

The phosphate depletion data consists of 988 DE genes, of which 67 are predefined regulators comprising DNA binding proteins and proteins capable of regulating gene expression indirectly such as kinases (see Section 1 of Supplementary Material). The regulators show a wide spread of correlation (Supplementary Fig. S6a); we expect interference to be only important among the highest correlating regulators and thus only examine the correlation threshold in the interval (0.7, 1). We grouped the 67 regulators into highly correlated sets by a hierarchical clustering method (Supplementary Fig. S6b). The merge points in the tree are the values of ω at which the network N(ω,ϕ) is determined. We used GRENITS to infer the casual network on all 988 genes (see Section 2 of Supplementary Material). Interference was then analysed on the merge points in 0.7<ω<1 across the link probabilities ϕ=0.4,0.55,0.6,0.65,0.7 following the method in Section 3. All these *ϕ* are in the tail of the posterior link probability distribution (Supplementary Fig. S4a) and significantly larger than the posterior estimate for random links, ρ=0.0266±0.0010 (distribution standard deviation). Therefore, links on all these thresholds demonstrate evidence of causal regulation at varying levels of significance.

**Fig. 2.**
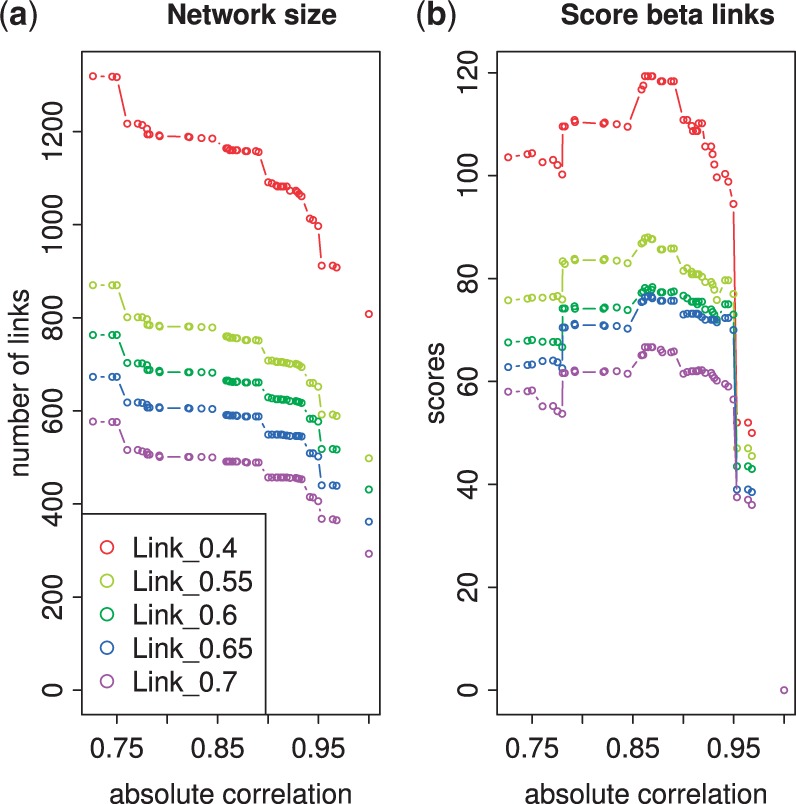
Analysis of the β-link composition of the *S.coelicolor* phosphate depletion ICN with respect to link *ϕ* and correlation ω thresholds. The total number of links (**a**) and the β-link network score (**b**) for networks N(ω,ϕ) are plotted against the absolute correlation threshold ω between regulators for link thresholds ϕ=0.4, 0.55, 0.6, 0.65, 0.7, see legend. At the correlation threshold ω=1, the network comprises α-links only determined from the posterior link probabilities

An analysis of the ICN is shown in Figure 2. Because the number of α-links remains constant and, typically, β-links are added to the network, the total number of links in the network increases on average with respect to increasing clustering stringency (decreasing ω) for all *ϕ* (Fig. 2a). The β-link network score indicates that levels of interference are initially strong but decrease as the cluster threshold falls <0.85. Thus, the score has a maximum around 0.86–0.90 for all the link probability thresholds (Fig. 2b), and therefore, the optimum $\omega^{*}(\phi )$ is fairly robust to changes in the link threshold *ϕ*. Interference is fairly uniform across link strength with 30–40% of links being recovered at the optimum correlation threshold $\omega^{*}(\phi )$ for each given *ϕ*.

**Fig. 3.**
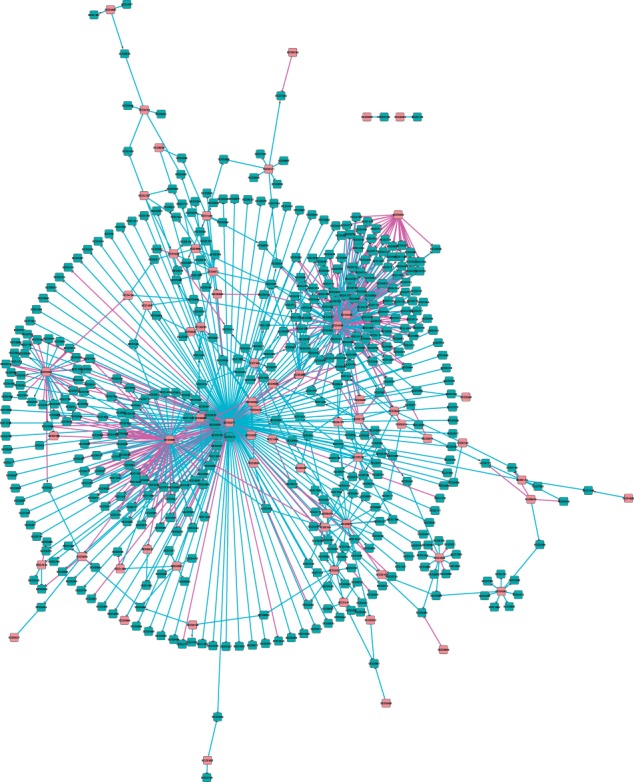
Cytoscape visualization of the *S.coelicolor* phosphate depletion optimal ICN, N(0.865,0.55). Nodes are regulators (pink) and (non-regulator) target genes (blue). There are 498 α-links (cyan) and 259 β-links (magenta). The T-arrow at the end of a link denotes inhibition, and an arrow denotes activation. Link length has no meaning. The network has a large hub around SCO3217 (*cdaR*), targets making up the large diameter, interferring with the close proximity hub SCO5881 (*redZ*), to the left, and with another small hub SCO4425, positioned far left; these three regulators are clustered together at ω=0.865. The second interfering cluster is the Pho regulon hub complex around SCO4228, SCO4229, SCO4230, merged hubs on top right; again these three regulators are clustered at ω=0.865. There are five smaller hubs, SCO4261, SCO4908, SCO5877, SCO7516 and SCO7463, lower right. Zoom into figure for detail or see cytoscape file (Supplementary Material)

As discussed in Section 3, the link threshold should be greater than 1/(2−ρ)=0.507 for a full interference assessment. To examine ICN structure and the potential biological importance of the inferred links, we choose ϕ=0.55 for illustration, much larger than ρ (the probability of random links) and close to the minimum threshold for interference detection. The β-link network score is maximized (for ϕ=0.55) at ω=0.865 (the highest absolute correlation with the maximum score) (Fig. 2b). The network N(0.865,0.55) has a main hub centred on SCO3217, the calcium-dependent antibiotic regulator *cdaR* and a smaller Pho regulon hub around PhoURP (Fig. 3). There are 259 recovered β-links of 757 links in the ICN, i.e. regulator interference caused a loss of 34% of links, 103 in the *cdaR* hub, interfering primarily with *redZ* (SCO5881), the principal regulator of the undecylprodigiosin antibiotic gene cluster (with a correlation of 0.95), and 125 in the *phoURP* hub, a result of cross-interference between *phoU*, *phoR* and *phoP* (correlation coefficients > 0.93) with 69, 32 and 24 β-links, respectively.

Interference to a common target is highly heterogeneous across regulators within a cluster. For illustration, classify targets for clustered regulators according to the type of links in the ICN, ranging from sole α targets (one regulator is an α-link, other regulators lack a link), mixed α/β targets (at least one α- and β-link), partial β targets (at least one β-link but not all β, and no α) and complete β targets; these respective classifications have increasing levels of mutual interference. The joint targets between SCO3217 and SCO5881 in ICN N(0.95,0.55) then show a diversity of classes, with the absolute target-regulator (time shifted) correlation predicting the target classification (Supplementary Fig. S9). Specifically, the sole α targets of SCO3217 have a higher absolute correlation with SCO3217 than with SCO5881, and *vice versa*, while complete β targets have closer correlation coefficients. The degree of mutual interference also shows variation (Supplementary Fig. S10); the complete β targets are evenly weighted with similar conditional link probabilities, whereas the partial β-links have greater differences in their conditional link probabilities. Results are similar for the optimal ICN but more complex because of the three-way interference between SCO3217, SCO5881 and SCO4425. Interference is thus heterogeneous within the target base of clustered regulators.

To assess levels of accuracy of our recovered β-links, we examined the 24 targets predicted for PhoP [seven are in the same operon as defined by (Charaniya *et al.*, 2007)]; in the uncorrected network, *phoP* had no targets, whereas *phoU* and *phoR* had 19 and 69, respectively at ϕ=0.55. It is highly likely that most of these targets are actually regulated by PhoP, the principle regulator of the phosphate response (Martin *et al.*, 2012; Rodriguez-Garcia *et al.*, 2007). We examined two criteria, firstly, whether these targets had distinctly different profiles under *phoP* knock out (Thomas *et al.*, 2012; Supplementary Figs S11 and S12), and whether there was evidence of a PHO box upstream of the gene, the binding site for PhoP (motif GTTCA). The first criteria showed that five predicted targets were significantly altered at the 0.5% level (SCO1196, SCO3899, SCO4726, SCO5390, SCO6753), and three others at 5% (Supplementary Table S3). The second criteria indicated that five targets (SCO3899, SCO4545, SCO4653, SCO4726, SCO6753) and the operon (seven genes) have strong evidence of a PHO box or a dyad, (Multiple EM (Expectation Maximization) for Motif Elicitation (MEME) score >10, resp. 6); five genes were in both categories (Supplementary Table S3). Close examination of the transcriptional profiles shows that many have a discernible difference in the dynamics across phosphate depletion; typically, ΔphoP shows slower changes in gene expression. All those with a PHO box show this behaviour. Thus, >50% of predicted targets have supporting evidence of a PhoP binding site (Supplementary Table S3). The two genes SCO1196 and SCO5390 underwent a dramatic change in expression, but there was little evidence of the presence of a PHO box. They may therefore be indirect targets or possess functional but altered PHO boxes. Together, this provides strong evidence that correcting for interference is essential because otherwise important targets may be missed. All of the PhoP targets were lost in absence of an interference correction in this case.

### 4.2 Example 2: glutamate depletion causal network

We examined the ICN for a second *S.coelicolor* dataset under glutamate depletion (Waldvogel *et al.*, 2011), a treatment that also gives rise to the synthesis of RED and ACT antibiotics. The data comprises 945 DE genes including 59 predefined DE regulators (see Section 1 of Supplementary Material). The absolute correlation between these regulators and the corresponding clustering tree (Supplementary Fig. S7) are similar to those in the phosphate depletion experiment (Supplementary Fig. S6). Under the same analysis as above, the optimal ICN occurred at a similar clustering threshold, ($\phi =0.55,\omega^{*}(0.55)=0.859$; Supplementary Figs S13 and S14) and comprises 283 links of which 35 are β-links, i.e. there is a substantially lower level of regulator interference in this dataset with an estimated loss of 12% by regulator interference. Thus, despite similar correlations among the regulators and a similar posterior link probability distribution (Supplementary Fig. S4b) to that in the phosphate depletion experiment, regulator interference, although significant at 35 β-links, was reduced. This may be because of the different network structure; under phosphate depletion the network comprises distinct separated large hubs, Fig. 3, whereas under glutamate depletion the network is more dispersed among a number of smaller hubs, Supplementary Fig. S14.

### 4.3 Example 3: *Arabidopsis* circadian clock network

**Fig. 4.**
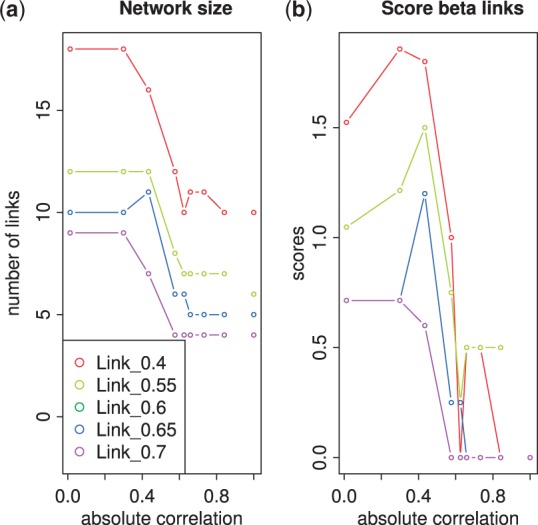
Analysis of the ICN β-link composition with respect to link *ϕ* and correlation ω thresholds for the *Arabidopsis* circadian clock data. The total number of links (**a**) and the score of β-links (**b**) for networks N(ω,ϕ) are plotted against the absolute correlation threshold ω between regulators for link thresholds ϕ=0.4, 0.55, 0.6, 0.65, 0.7, see legend

**Fig. 5.**
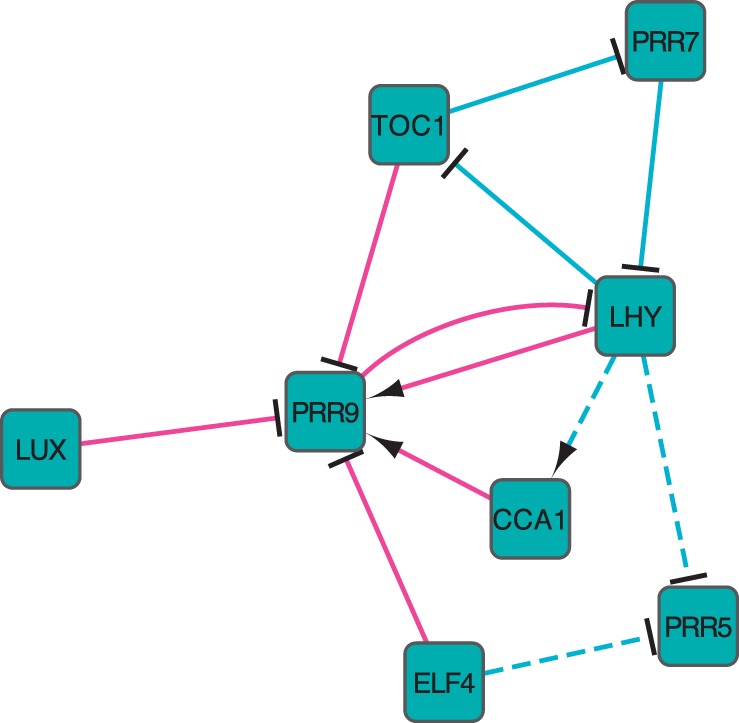
Inferred *Arabidopsis* circadian clock ICN showing α-links (cyan) and β-links (magenta). Solid lines indicate predictions consistent with the latest experimental data and model (Pokhilko *et al.*, 2013), while the dashed lines indicate predictions that are not thus far supported by current literature. Arrows indicate activation and the T-arrows inhibition

The *Arabidopsis* circadian clock is one of the most well-established biological networks containing both transcriptional and translational regulation. We used transcriptome data comprising 10 regulators (Windram *et al.*, 2012), every 2 h up to 48 h, 24 time points in total. There are four biological replicates at each time point allowing the replicate causal linear model with Student noise in GRENITS to be used for network inference (Morrissey *et al.*, 2010). The absolute correlation between these regulators and the corresponding clustering tree are shown in Supplementary Fig. S8. The ICN N(ω,ϕ) was constructed following the method in Section 3 (Fig. 4) and displayed similar trends to the two previous larger networks (Fig. 2; Supplementary Fig. S13). We constructed the optimal ICN for ϕ=0.55 (Fig. 5); the lowest link threshold for a full interference assessment is 1/(2−ρ)=0.543 in this case (Supplementary Fig. S5).

To validate our method, we compared our reconstructed network with the *Arabidopsis* circadian clock model in (Pokhilko *et al.*, 2013), defining the ground truth network (Supplementary Table S4). We inferred 12 links, which consist of 6 α-links and 6 β-links (Fig. 5). In particular, all the β-links are correct, whereas only three of the α-links are correct. The precision of our ICN prediction is thus 75%, subject to the degree to which the network is actually known. Furthermore, an analysis of the ICNs across link threshold *ϕ* shows that the optimal ICN always has maximal precision for link thresholds ϕ=0.55 and above (Supplementary Fig. S15). Finally, a comparison between NIACS and Murphy’s Bayes Net Toolbox (Murphy and Mian, 1999) shows that our method has much higher precision (Supplementary Table S5).

## 5 DISCUSSION

In this article, we have proposed a method for dealing with the problem of regulators with similar dynamics suppressing causal link signals in the auto-regression model Equation (1). By analysis of the level of interference between highly correlated regulators (computed from the posterior conditional link probabilities), we are able to correct for link suppression by near identical regulators, and thus recover regulator links that would otherwise be lost because their posterior link probabilities are reduced below threshold. We defined an ICN $N(\omega^{*},\phi )$ that includes as a subnetwork the original network N(1,ϕ), i.e. our method only adds links lost by interference. The ICN is defined at the value of the correlation threshold $\omega^{*}$ where interfering regulators are judged to be optimally clustered as measured by a score that sums over weighted β-links; our score punished clustering of inconsistent regulators through a lack of mutual interference or no interference.

Our approach avoids a subjective subselection of the set of predefined regulators, i.e. all regulators should be included in the inference in absence of prior information, and, by a post-processing step, interference is corrected. In effect, we are assuming that there are potentially multiple regulators for a target, but network inference is hampered by insufficient dynamic data to separate signals from (individual) regulators with similar dynamics. Determining whether there are two true regulators as opposed to one is not possible without additional experimental data, e.g. experiments under conditions where the regulators have distinct dynamics or conducting experiments with strains where either regulator is expressed under an inducible promotor. Our method crucially prevents loss of both regulators from the inferred network. We demonstrated that the recovered β-links for PhoP and the *Arabidopsis* circadian clock are functional, with precision at least as good as that for the predicted α-links. In a comparison against other network inference methods on simulated data (DREAM4), GRENITS is already highly competitive, while performance is further improved when coupled with NIACS to correct for regulator interference, the area under precision recall curve increasing on average by 10% (Supplementary Table S2). Most of the improvement is in the crucial high confidence predictions (Supplementary Fig. S3). NIACS outperformed the other methods in 10 of 14 cases.

For the ICN $N(\omega^{*},\phi )$, we assign the link probability as the maximum of the posterior link probability and the conditional probability (if applicable). However, we do not expect the conditional probability $\pi (\gamma_{ij}=1\;|\;D,\gamma_{ik}=0,\forall k\in CR^{s}(\omega^{*})\setminus \{j\})$ to truly reflect the probability of regulator *j* regulating *i*, as this is dependent on the clustering set $CR^{s}(\omega^{*})$. In essence, if there are multiple regulators, they all affect the dynamics (if on), and a true measure of their regulatory effects is in the presence of the other regulators. Therefore, we recommend that β-links arising from interference remain distinguished because their probabilities are corrected and thus a direct comparison with α-links may be misleading. However, the relative weighting within a cluster of regulators according to their conditional posterior probabilities is a reflection of the evidence in the data as to the identity of the true regulator. The identity of this true regulator may also be discerned by using additional experiments or gene annotations. These data can also be used as prior information in GRENITS restricting allowable links, thereby removing some of the regulator ambiguity.

Our analysis on three real datasets demonstrated that interference is not simply a function of regulator correlation. In the phosphate depletion causal network (Example 1), for two highly correlated regulators, we found a spread of interference among their targets comprising a set of targets on which interference is mutual, whereas other targets have only a sole regulator despite their high correlation; interference is thus heterogeneous on the set of targets. These observations mean that interference must be analysed on a per experiment, per target basis and the optimal correlation threshold determined in each case.

In addition to regulator interference, we also observed another phenomenon in our networks, whereby two (or more) regulators enhance the causal signals of each other; specifically, the regulators have elevated posterior link probabilities in the presence of each other. We define pairwise *synergistic* regulators *j*, *k* on target *i* as regulators with $\pi (\gamma_{ij}=1\;|\;D),\pi (\gamma_{ik}=1\;|\;D)>\phi$, $\pi (\gamma_{ij}=1\;|\;D,\gamma_{ik}=0),\pi (\gamma_{ik}=1\;|\;D,\gamma_{ij}=0)<\phi$ (this definition can be easily generalized to the case of multiple regulators). Causal signal synergy between regulators was rare, with 3, 3 and 1 synergistic regulators in Examples 1, 2 and 3, respectively (Supplementary Table S6). We emphasize that neither interference nor synergy between the causal signals implies that a mechanistic interference/synergy exists. Link suppression/enhancement from interference/synergy in our context arises because of similarity (correlation) in the regulator dynamics.

Interference between dynamically similar regulators in causal network inference is a general problem. It will occur in any statistical hypothesis testing methodology for the model in Equation 1. For instance, as the weights *B_ij_* in Equation 1 are suppressed under interference, and their variance increases under a lack of (*a posteriori*) identifiability, any test for a non-zero coefficient will be subject to loss of highly correlated regulator links (see Section 4.2 of Supplementary Material). In this article, we have provided a methodology to recover missing (suppressed) links within a Bayesian context through a post-processing of the network posterior samples.

## Supplementary Material

Supplementary Data
