# Supplementary material for: Correcting for link loss in causal network inference caused by regulator interference
Source: Bioinformatics. 2014 Jun 19;30(19):2779–86. doi: 10.1093/bioinformatics/btu388 (PMC4173021; doi:10.1093/bioinformatics/btu388)
Supplement: Supplementary Data [file supp_30_19_2779__index.html]

Correcting for link loss in causal network inference caused by regulator interference — Correcting for link loss in causal network inference caused by regulator interference — Correcting for link loss in causal network inference caused by regulator interference — Supplementary Data 

# Correcting for link loss in causal network inference caused by regulator interference

## Supplementary Data

files

**Files in this Data Supplement:**

- Supplementary Data - zip file
