# Supplementary material for: Correcting for link loss in causal network inference caused by regulator interference
Source: Bioinformatics. 2014 Jun 19;30(19):2779–86. doi: 10.1093/bioinformatics/btu388 (PMC4173021; doi:10.1093/bioinformatics/btu388)
Supplement: Supplementary Data [file supp_btu388_suppl_data.zip › SupplementaryMaterials.pdf]

Supplementary Materials: Correcting for link loss in causal network inference caused by regulator interference

Ying Wang [1], Christopher A. Penfold[1], David A. Hodgson [2], Miriam L. Gifford [2], and Nigel J. Burroughs [1*]
[1]Warwick Systems Biology Centre and [2]School of Life Sciences,
University of Warwick, Coventry CV4 7AL, UK
*Corresponding author. N.J.Burroughs@warwick.ac.uk

March 24, 2014

Contents

1 Experimental Data **1**
1.1 Phosphate Depletion Data 1
1.2 *phoP* Mutant Data 2
1.3 Glutamate Depletion Data 2
1.4 Arabidopsis Circadian Clock Data 2

2 MCMC (GRENITS) **2**
2.1 Conjugate Priors of Linear Model 3
2.2 GRENITS Parameters of Linear Model 3
2.3 Convergence Analysis 4

3 Comparing network inference methods **5**

4 Supplementary Proofs **8**
4.1 Multiple identical regulators 8
4.2 Interference in general regression 8

5 Supplementary Figures **10**

6 Supplementary Tables **24**

7 List of cytoscape files **26**

1 Experimental Data

1.1 Phosphate Depletion Data

This gene expression data set is from a time-series experiment performed on *S. coelicolor* under phosphate depletion (GEO: GSE18489/F199), (Nieselt et al., 2010). *S. coelicolor* was germinated from spores in a stirred aerated fermentor with limited phosphate; phosphate in solution was exhausted at 35hrs (Wentzel et al., 2012). Samples were taken every hour from 20 hrs to 44 hrs, and then every two hours until 60 hrs; the sample at 25 hrs was missing in the original dataset. The 32 (Affymetrix) arrays were processed using RMA with loess normalisation. 988 differentially expressed

(DE) genes were obtained using the BATS package (Angelini et al., 2008). A list of transcriptional regulators of *S. coelicolor* was determined from functional annotations and included known regulator families (AraC, ArsR, TetR, Sigma factors, anti-Sigma factors etc), DNA binding proteins and potential phosphorylation mediated regulators such as 2 component systems and kinases. Of the 988 DE genes in this experiment, 67 are predefined regulators. In our causal network construction we used gene expression data for the 988 DE genes at 19 time points from 26 hrs to 44 hrs with 1 hr resolution.

1.2 *phoP* Mutant Data

Data is from a time-series performed on a *S. coelicolor mutant*, INB201 where the *phoP* gene is disrupted and non-functional (GEO: GSE31068, Affymetrix platform), (Thomas et al., 2012). This strain, denoted ΔphoP in the main text, was grown under similar conditions to the wild type above (GEO: GSE18489/F199); phosphate was exhausted at 41 hrs in this strain. Samples were taken every hour from 23 hrs to 36 hrs and from 41 hrs to 48 hrs, every half hour from 36 hrs to 41 hrs, and every two hours from 48 hrs to 60 hrs; the sample at 34 hrs was missing in the original data set. The 36 arrays were processed using RMA as above.

1.3 Glutamate Depletion Data

Data is from a time-series experiment performed on *S. coelicolor* under glutamate depletion (GEO: GSE30569, Affymetrix platform), (Waldvogel et al., 2011); glutamate was limiting and was exhausted at 35 hrs, (Wentzel et al., 2012). 31 samples were taken: at 20 hrs, at one hour resolution from 24-32 hrs and 40-42 hrs, half an hour resolution from 32-40 hrs, and at 44 hrs, 46 hrs, 54 hrs and 58 hrs; data for the time point 34 hrs was missing in the original data set. The 31 arrays were processed using RMA as above and DE genes were determined using BATS. There are 945 DE genes including 59 predefined regulators. In our analysis, we used the gene expression data for the 945 DE genes at 19 time points from 24 hrs to 42 hrs with 1 hr resolution except that 34.5 hrs was used for the missing 34 hrs time point.

1.4 Arabidopsis Circadian Clock Data

Data is from a time-series experiment performed on *Arabidopsis thaliana* for both the *B. cinerea*-infected and mock-inoculated leaves (GEO: GSE29642, CATMA platform), (Windram et al., 2012). In the original experiment, 192 samples were taken at two hours resolution from 2 hours to 48 hours, with four biological replicates for each time point. Arrays were normalised with a modification of the MAANOVA package (Wu et al., 2003; Breeze et al., 2011). In our analysis, we used the mock-inoculated gene expression data (four biological replicates at each time point) for the 10 circadian clock genes: LHY, CCA1, PRR5, PRR7, PRR9, TOC1, GI, LUX, ELF3 and ELF4.

2 MCMC (GRENITS)

In this section, we state the (conjugate) priors used in the Bayesian inference (GRENITS algorithm; http://www.bioconductor.org/packages/2.12/bioc/html/GRENITS.html) and the run parameters for the linear model used on the *S. coelicolor* data sets. We demonstrate convergence on the phosphate depletion data set (Example 1). Since there are four biological replicates at each time point for the gene expression data of *Arabidopsis* circadian clock genes, we used the replicate causal inference model in GRENITS with Student noise (Morrissey et al., 2010); all the parameters are at their default values in GRENITS.

2.1 Conjugate Priors of Linear Model

The prior is given by

$$\pi(\theta) = \pi(\rho)\left(\prod_{i=1}^{G}\pi(\mu_i)\pi(\lambda_i)\Big(\prod_{j=1}^{G}\pi(\gamma_{ij}\mid\rho)\pi(B_{ij})\Big)\right), \tag{1}$$

where

$$\begin{aligned}
\pi(\mu_i) &= N(\mu_i \mid 0, 4),\\
\pi(\lambda_i) &= Ga(\lambda_i \mid 2, 0.01),\\
\pi(\gamma_{ij}\mid\rho) &= Ber(\gamma_{ij}\mid\rho),\\
\pi(\rho) &= Be(\rho \mid 0.5, 0.5),\\
\pi(B_{ij}) &= N(B_{ij} \mid 0, 4).
\end{aligned}$$

2.2 GRENITS Parameters of Linear Model

Table S1: Parameters used for inference in the linear model of GRENITS on $D^{1\times phoP}$, $D^{2\times phoP}$, phosphate depletion data (Example 1) and glutamate depletion data (Example 2).

samples	burnin	thinning rate	c	d	sigma.s	a	b	sigma.mu
2×10^6	2×10^5	10^2	0.5	0.5	2	2	0.01	2

2.3 Convergence Analysis

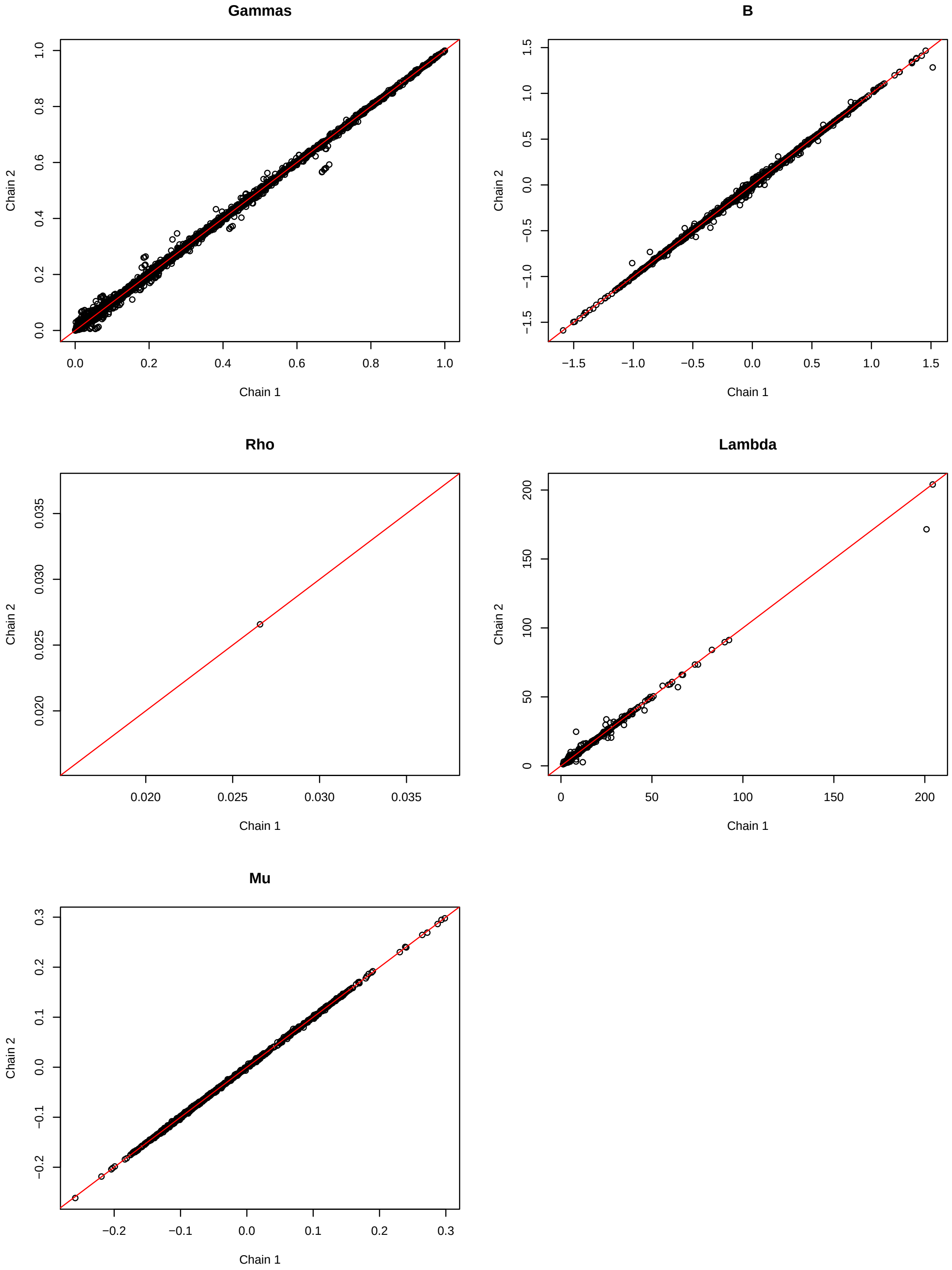

Figure S1: Convergence plots from GRENITS for the phosphate depletion data, Example 1. The posterior means (∘) of each variable ($\{(\gamma_{ij}), (B_{ij}), \rho, (\lambda_i), (\mu_i)\}$) are compared between two MCMC chains of GRENITS. Most posterior means are near identical (∘ lying on the red diagonal line). This confirms that the GRENITS sampler mixes well. Run parameters as Table S1.

3 Comparing network inference methods

We partially replicated the comparison analysis from (Penfold and Wild, 2011), using the 100-gene simulated networks from the DREAM4 challenge, available from the DREAM project homepage (www.the-dream-project.org/category/challengesdream/dream4). Each data set comprises 10 perturbation *in silico* time series experiments of 21 time points of a known 100 gene network. We inferred the networks from 1 experiment (1 time series), 3 experiments (combination of 3 time series) and the full data set (all 10 time series). Regulator interference will fall as more data sets are included since regulators are less likely to have similar dynamics under an increasing number of conditions. Example gene correlations between putative regulators (all genes are potential regulators) for these sets are shown in Supp. Figure S2.

As GRENITS does not allow for the use of multiple time-series representing different experimental conditions, the time-series were concatenated. The concatenated time-series has inconsistent time-steps where the individual time-series are joined, although this represents only a small percentage of the time-steps (4%). We used the linear model in GRENITS throughout. For NIACS, we examined the optimal correlation threshold ω across a range of link thresholds ϕ. The optimal ω was fairly stable, so we used the optimal threshold for $\phi = 0.6$ throughout, i.e. for the optimal ω (within 0.7-0.9 depending on the data set), we constructed the corrected link probabilities (max(PP,CP) as text) and based the assessment statistics on the network with these link probabilities. Two other methods were chosen for comparison, representing the categories of ordinary differential equations (TSNI; (Bansal et al., 2006)) and Dynamic Bayesian Networks (G1DBN; http://cran.r-project.org/web/packages/G1DBN/index.html); see (Penfold and Wild, 2011) for details on the models and the data sets. As with GRENITS, neither of these additional methods were designed with multiple perturbed time series data in mind, and time series were therefore concatenated together.

Table S2: Area under Precision-Recall curve for different methods (4 decimal places). The best performing method per experiment is shown in bold. The value for random networks is 0.002.

Profiles	TSNI	G1DBN	GRENITS	GRENITS+NIACS
1	0.0443	0.0362	0.0388	**0.0493**
2	0.0251	**0.0650**	0.0549	0.0584
3	0.0583	**0.0697**	0.0633	0.0634
4	0.0456	0.0430	0.0519	**0.0593**
5	0.0452	0.0445	0.0606	**0.0698**
6	0.0315	0.0310	0.0494	**0.0706**
7	0.0448	0.0645	0.0680	**0.0764**
8	0.0457	0.0488	0.0546	**0.0564**
9	0.0316	**0.0577**	0.0345	0.0389
10	0.0521	0.0581	0.0621	**0.0630**
1,2,3	0.0632	0.1236	**0.1508**	0.1501
4,5,6	0.0471	0.1293	0.1331	**0.1402**
7,8,9	0.0371	0.0974	0.1175	**0.1205**
1-10	0.0755	0.0167	0.2603	**0.2701**

To assess the quality of the predictions the area under the precision-recall curve (AUPR) was calculated (Supp. Table S2) and example ROC curves are also shown in Supp. Figure S3. In general, GRENITS is a high performing method, yielding better AUPR scores than TSNI or G1DBN in 7 out of 10 of the single time series datasets, with G1DBN best in three cases. For the combined datasets, GRENITS was better in all four situations, with G1DBN performing better than TSNI in 3 out of 4 cases (it should be noted that G1DBN, which generally performs well, performed poorly when all 10 perturbations were concatenated and might therefore be more sensitive to this way of combining the data). NIACS appeared to be consistently better than GRENITS, with higher AUPR scores in 13 of the 14 datasets, and was the best method in 7 out of 10 single experiments, 2 out of the 3 triple experiments and the best in the combined data set, Supp. Table S2. Most of this

improvement occurs in the high confidence predictions (high ϕ), as characterised by the left hand side of the ROC curve, Supp. Figure S3, the ROC curve for NIACS (black lines) is nearly always found shifted up and to the left compared to GRENITS (blue lines).

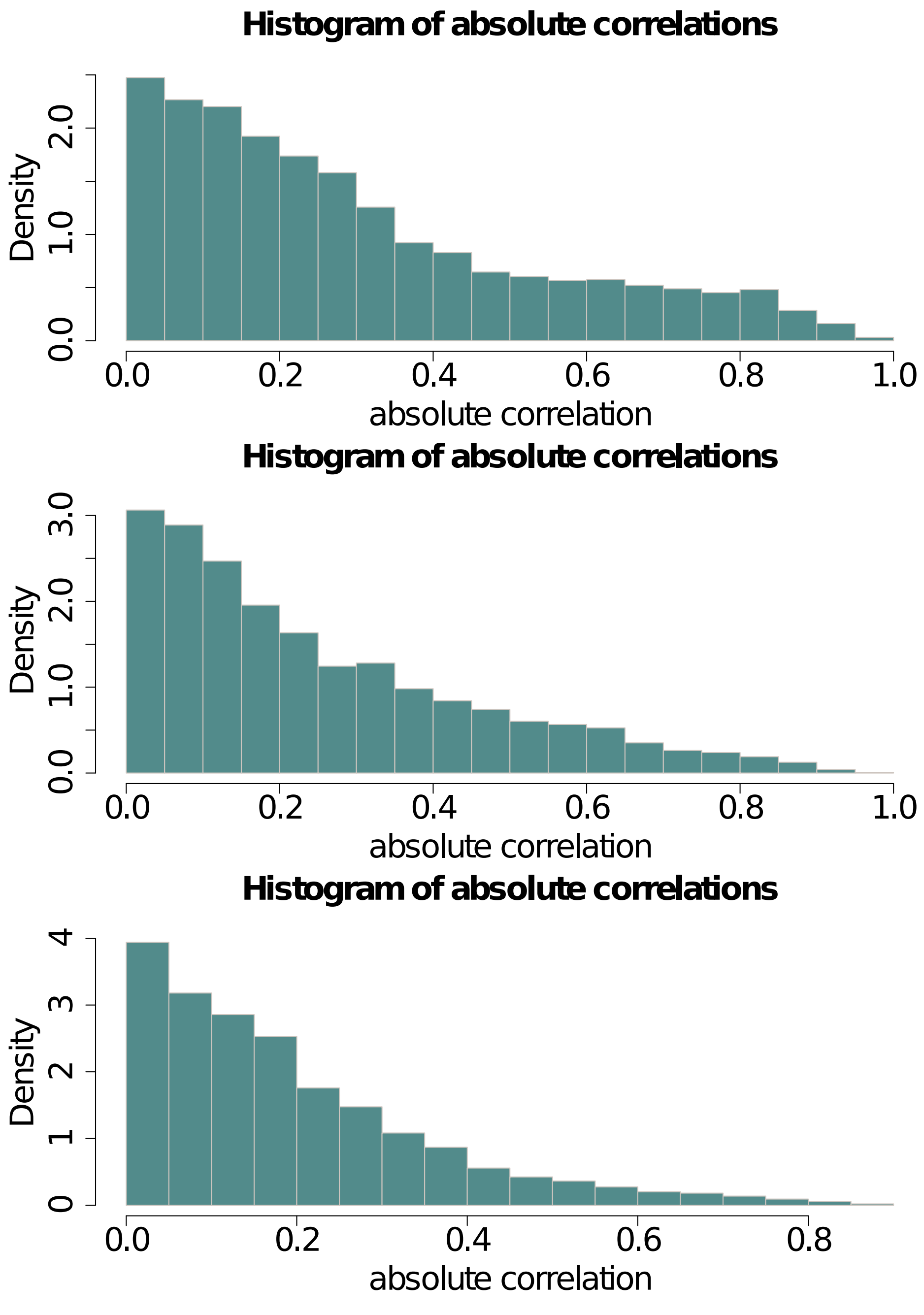

Figure S2: Example of correlation between all putative regulators in the DREAM4 100-gene datasets. Correlations in a single representative perturbation time series, Top, in three time series combined, Middle, and all 10 time series, Bottom. As more time series are combined the overall correlation and therefore overall interference between putative regulators decreases. Correlation structure is distinct from real data sets, compare to Fig. S6, S7.

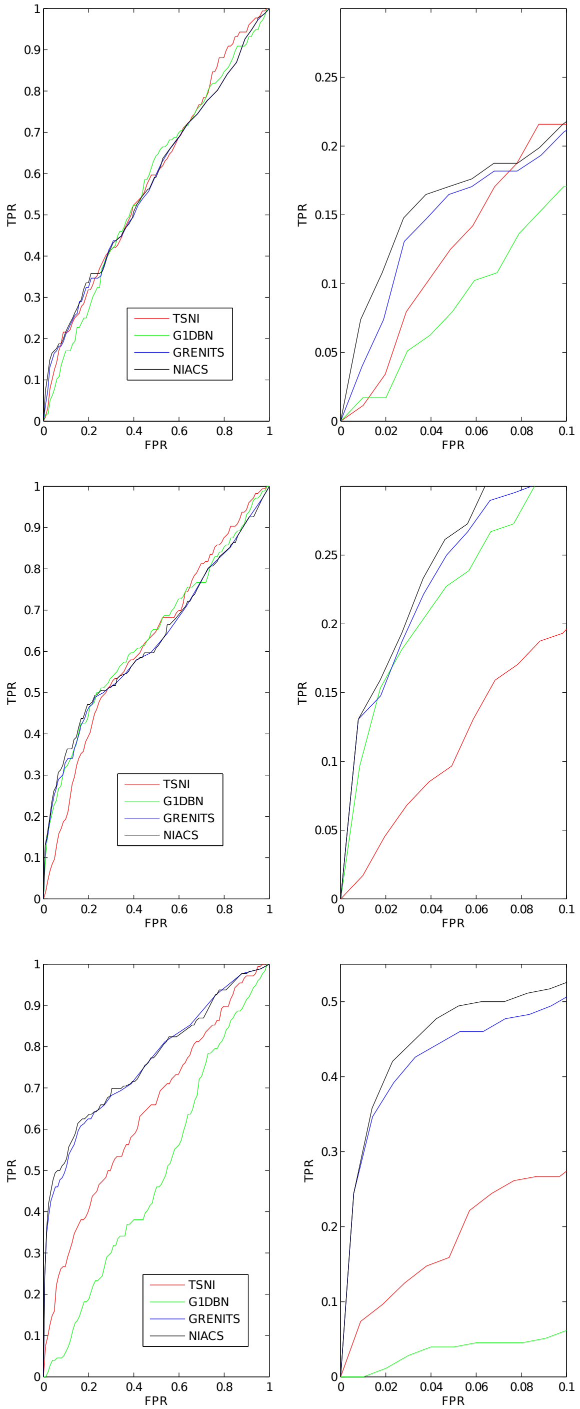

Figure S3: Example ROC curves for different methods using different combinations of data. Shown are the complete ROC (left) and the high confidence regions (right), i.e. where the false positive rate is < 0.1. Three examples are included: a single time series dataset (Top); a combination of three time series (Middle); the combination of all 10 time series (Bottom). In general, NIACS performs better than GRENITS and other methods, particularly at the high confidence regions (see right hand pannels).

4 Supplementary Proofs

4.1 Multiple identical regulators

For a set S_i of regulators of target i with identical expression, $|S_i| = n$, the analysis in section 2.1 of the main text generalises to give,

$$\pi(\gamma_{ij} = 1 \mid D, \gamma_{ik} = 0,\ \forall k \in S_i \setminus \{j\}) \approx \frac{(1-\rho)^{n-1}\pi(\gamma_{ij} = 1 \mid D)}{1 - \frac{1-(1-\rho)^{n-1}}{\rho}\pi(\gamma_{ij} = 1 \mid D)}, \tag{2}$$

or

$$\pi(\gamma_{ij} = 1 \mid D) \approx \frac{\pi(\gamma_{ij} = 1 \mid D, \gamma_{ik} = 0,\ \forall k \in S_i \setminus \{j\})}{(1-\rho)^{n-1} + \frac{1-(1-\rho)^{n-1}}{\rho}\pi(\gamma_{ij} = 1 \mid D, \gamma_{ik} = 0,\ \forall k \in S_i \setminus \{j\})}. \tag{3}$$

A sketch of proof is as follows. Randomly choose m elements out of the set S_i, denoted U_i, and let $V_i = S_i \setminus U_i$. Let $f(m, n-m)$ denote the joint posterior distribution of $\{\gamma_{il}, l \in S_i\}$ with $\gamma_{iu} = 1$ for $u \in U_i$ and $\gamma_{iv} = 0$ for $v \in V_i$. For $m = 1, \cdots, n$, we have

$$f(m, n-m) \approx (\frac{\rho}{1-\rho})^{m-1} f(1, n-1),$$

where the approximate equality is due to the change of prior on certain elements of the matrix B as previously. Then we deduce that,

$$\begin{aligned}
\pi(\gamma_{ij} = 1 \mid D, \gamma_{ik} = 0,\ \forall k \in S_i \setminus \{j\}) &= \frac{f(1, n-1)}{1 - (n-1)f(1, n-1) - \sum_{m=2}^{n}\binom{n}{m} f(m, n-m)} \\
&\approx \frac{f(1, n-1)}{1 - \left(\frac{1}{\rho(1-\rho)^{n-1}} - \frac{1}{\rho}\right) f(1, n-1)}
\end{aligned}$$

We also obtain that,

$$\begin{aligned}
\pi(\gamma_{ij} = 1 \mid D) &= \sum_{k=0}^{n-1}\binom{n-1}{k} f(k+1, n-k-1) \\
&\approx \sum_{k=0}^{n-1}\binom{n-1}{k} (\frac{\rho}{1-\rho})^k f(1, n-1) \\
&= \left(1 + \frac{\rho}{1-\rho}\right)^{n-1} f(1, n-1).
\end{aligned}$$

Therefore, Equations 2 and 3 hold.

4.2 Interference in general regression

Interference will also occur in general regression problems. For simplicity, we compare two linear models. In Model 1, the linear regression model with Gaussian noise is formulated as follows:

$$y = cx + \varepsilon, \tag{4}$$

where x and y are variables with n observations $\{(x^1, y^1), \cdots, (x^n, y^n)\}$, c is a parameter with a prior distribution $N(0, \sigma_c^2)$, and ε follows the Gaussian distribution $N(0, \sigma_e^2)$. Then the posterior distribution of the parameter c is also a Gaussian distribution. The mean of c, denoted by M_c, is

$$M_c = \frac{\sigma_c^2(\sum_{i=1}^{n} x^i y^i)}{\sigma_e^2 + \sigma_c^2 \sum_{i=1}^{n}(x^i)^2}. \tag{5}$$

The variance of c, denoted by V_c, is

$$V_c = \frac{\sigma_c^2 \sigma_e^2}{\sigma_e^2 + \sigma_c^2 \sum_{i=1}^{n}(x^i)^2}. \tag{6}$$

In Model 2, the linear regression model with Gaussian noise is formulated as follows:

$$y = ax_1 + bx_2 + \varepsilon, \tag{7}$$

where x_1, x_2 and y are variables with n observations $\{(x_1^1, x_2^1, y^1), \cdots, (x_1^n, x_2^n, y^n)\}$, a and b are parameters with a joint prior distribution which is a two-dimensional Gaussian distribution with mean vector $\begin{pmatrix} 0 \\ 0 \end{pmatrix}$ and covariance matrix $\Sigma_p = \begin{pmatrix} \sigma_a^2 & 0 \\ 0 & \sigma_b^2 \end{pmatrix}$, and ε follows the Gaussian distribution $N(0, \sigma_e^2)$. The joint poster distribution of the parameters a and b is also Gaussian. Let $X = \begin{pmatrix} x_1^1 & \cdots & x_1^n \\ x_2^1 & \cdots & x_2^n \end{pmatrix}$. Let $\mathbf{y} = (y^1, \cdots, y^n)^\top$. Let $G = \sigma_e^{-2} XX^\top + \Sigma_p^{-1}$. Then the mean of the joint posterior distribution of the parameters a and b is $\sigma_e^{-2} G^{-1} X\mathbf{y}$, and the variance is G^{-1}.

We are interested in the case when the observations in Model 2 are duplicates of the observations in Model 1. That is, we assume $(x_1^1, \cdots, x_1^n)^\top = (x_2^1, \cdots, x_2^n)^\top = (x^1, \cdots, x^n)^\top$. Then the marginal posterior distribution of the parameter a is a Gaussian distribution. The mean of a, denoted by M_a, is

$$M_a = \frac{\sigma_a^2(\sum_{i=1}^n x^i y^i)}{\sigma_e^2 + (\sigma_a^2 + \sigma_b^2)\sum_{i=1}^n (x^i)^2}. \tag{8}$$

The variance of a, denoted by V_a, is

$$V_a = \frac{\sigma_a^2\sigma_e^2 + \sigma_a^2\sigma_b^2 \sum_{i=1}^n (x^i)^2}{\sigma_e^2 + (\sigma_a^2 + \sigma_b^2)\sum_{i=1}^n (x^i)^2}. \tag{9}$$

The marginal posterior distribution of the parameter b is a Gaussian distribution. The mean of b, denoted by M_b, is

$$M_b = \frac{\sigma_b^2(\sum_{i=1}^n x^i y^i)}{\sigma_e^2 + (\sigma_a^2 + \sigma_b^2)\sum_{i=1}^n (x^i)^2}, \tag{10}$$

identical to M_a except for the difference in the prior. Similarly, the variance of b, denoted by V_b, is

$$V_b = \frac{\sigma_b^2\sigma_e^2 + \sigma_a^2\sigma_b^2 \sum_{i=1}^n (x^i)^2}{\sigma_e^2 + (\sigma_a^2 + \sigma_b^2)\sum_{i=1}^n (x^i)^2}. \tag{11}$$

In order to compare the means and variances of the parameters we assume that $\sigma_a^2 = \sigma_b^2 = \sigma_c^2$. Then we obtain that $M_c > M_a = M_b$ and $V_c < V_a = V_b$. This demonstrates the presence of interference, in particular the evidence for the coefficients a, b to be non zero will be degraded relative to that for the single regressor c since their means are closer to zero and their variances increase.

5 Supplementary Figures

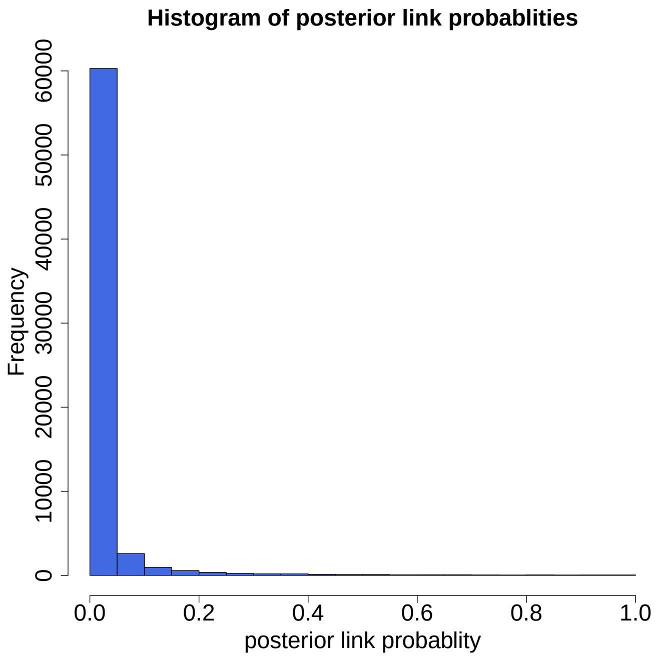

(a) Phosphate depletion

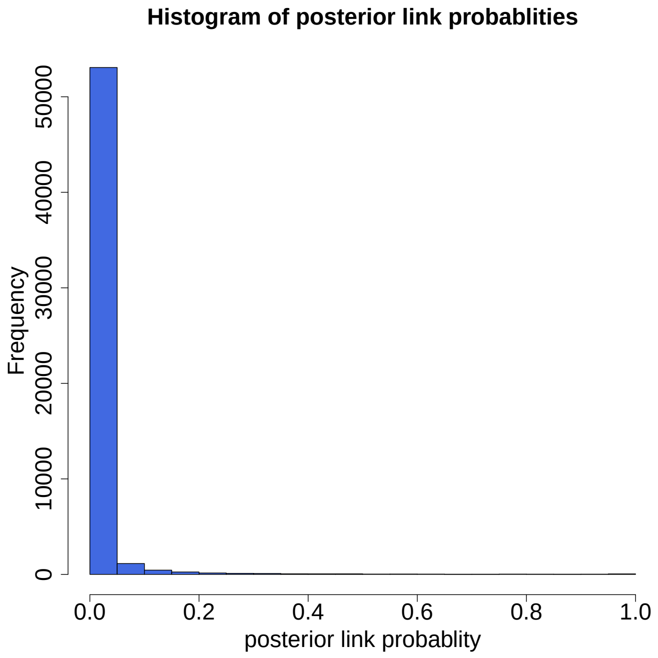

(b) Glutamate depletion

Figure S4: Histograms of the posterior link probabilities on (a) phosphate depletion data, Example 1, and (b) glutamate depletion data, Example 2. The distribution means are 0.0266 (distribution sd 0.0824) and 0.0157 (distribution sd 0.0648) respectively, close to the posterior means of the parameter ρ, 0.0266 (distribution sd 0.0010) and 0.0209 (distribution sd 0.0012) respectively. MCMC samples were generated with the GRENITS linear model, run parameters as Table S1.

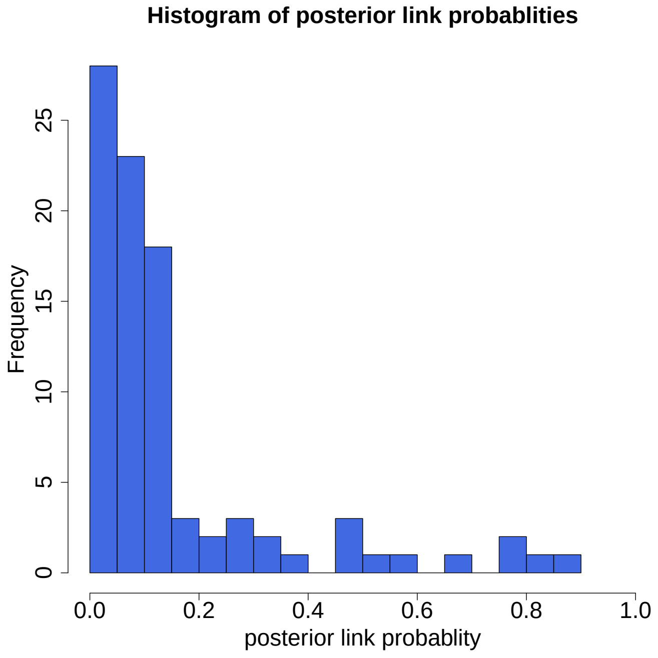

Figure S5: Histograms of the posterior link probabilities on *Arabidopsis* circadian clock data, Example 3. The distribution mean is 0.1549 (distribution sd 0.1926), close to the posterior mean of the parameter ρ, 0.1587 (distribution sd 0.0461). MCMC samples were generated with the replicate causal inference model in GRENITS with Student noise (Morrissey et al., 2010), run with the default parameters values in GRENITS.

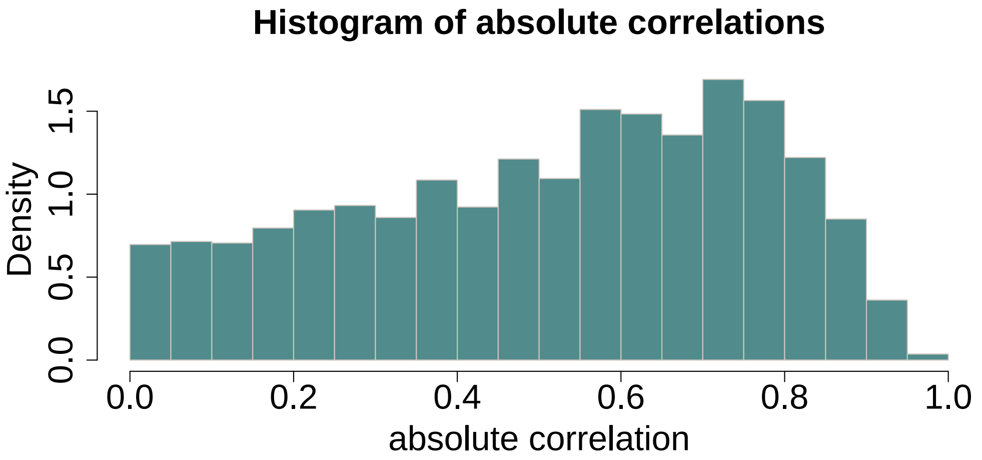

(a) Histogram of absolute correlations of regulators

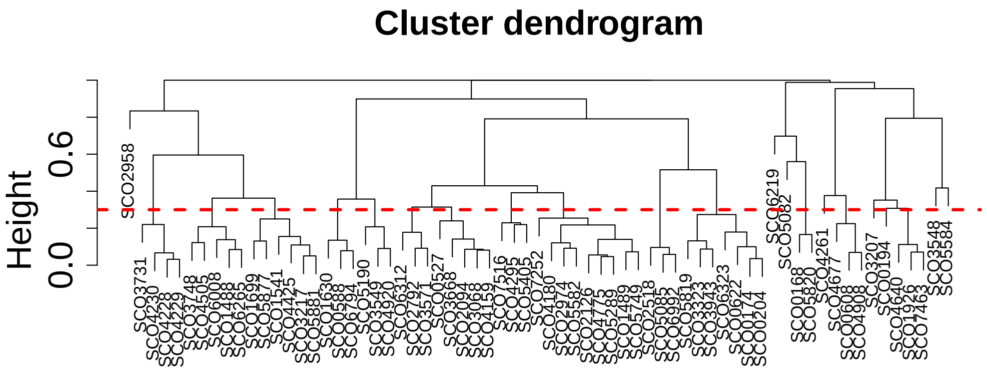

(b) Cluster dendrogram for the regulators

Figure S6: Correlations between regulators in the phosphate depletion data set, Example 1. (a) Histogram of absolute correlations of regulators; self correlations (=1) are excluded. (b) Cluster dendrogram for the regulators. The regulators are grouped using a hierarchical clustering method (R package "hclust"), where the distance between every two regulators is measured by one minus their absolute correlation and the agglomeration method is "complete". The horizontal red dashed line is at the height of 0.3. We only considered hierarchical clusters of regulators with merge heights below this line.

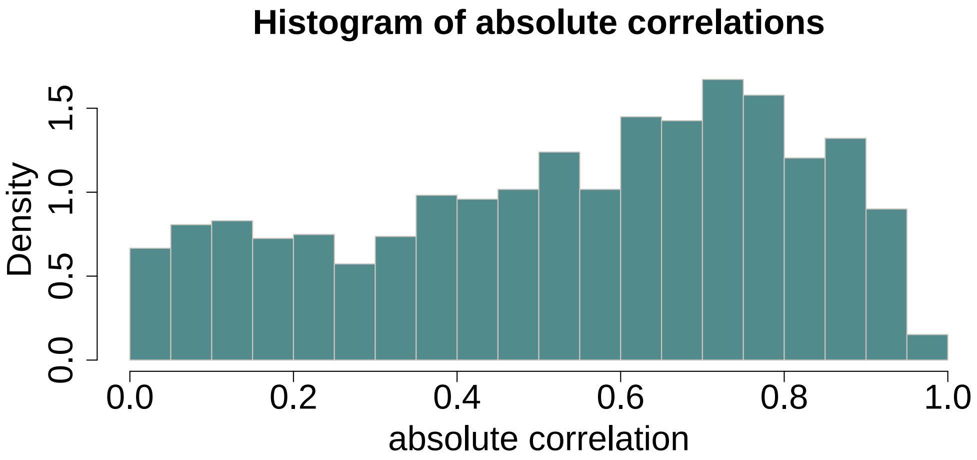

(a) Histogram of absolute correlations of regulators

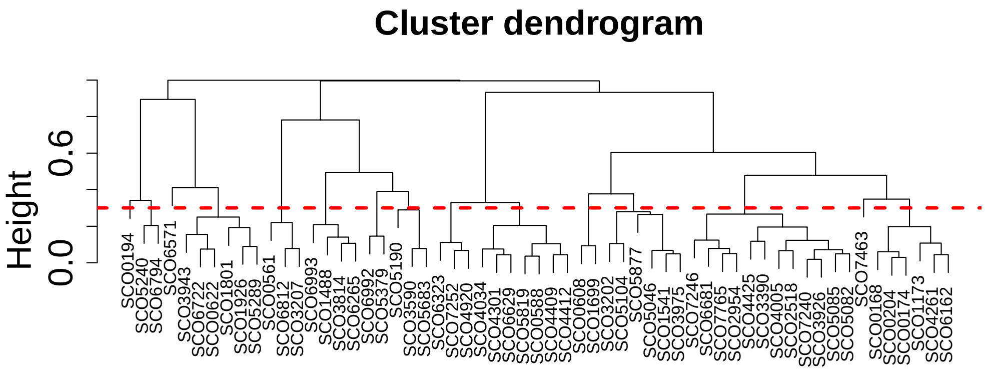

(b) Cluster dendrogram for the regulators

Figure S7: Correlations between regulators in the glutamate depletion data set, Example 2. (a) Histogram of absolute correlations of regulators; self correlations (=1) are excluded. (b) Cluster dendrogram for the regulators. The regulators are grouped using a hierarchical clustering method (R package "hclust"), where the distance between every two regulators is measured by one minus their absolute correlation and the agglomeration method is "complete". The horizontal red dashed line is at the height of 0.3. We only considered hierarchical clusters of regulators with merge heights below this line.

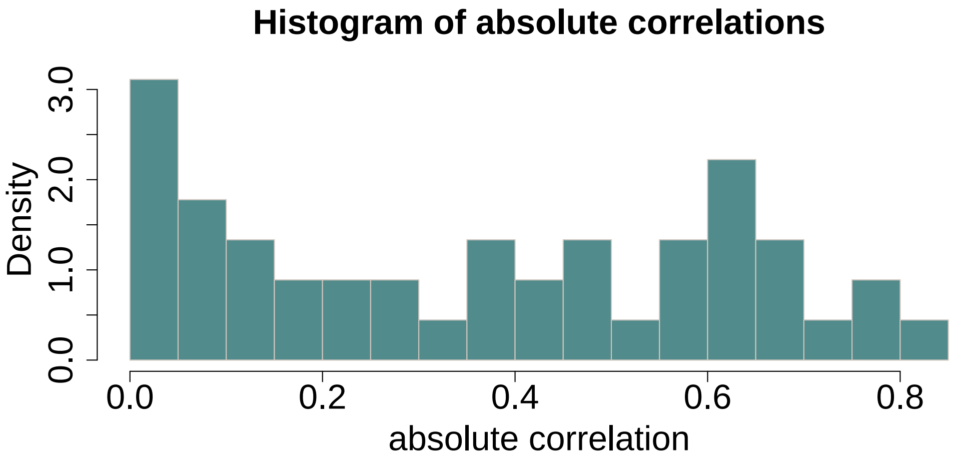

(a) Histogram of absolute correlations of regulators

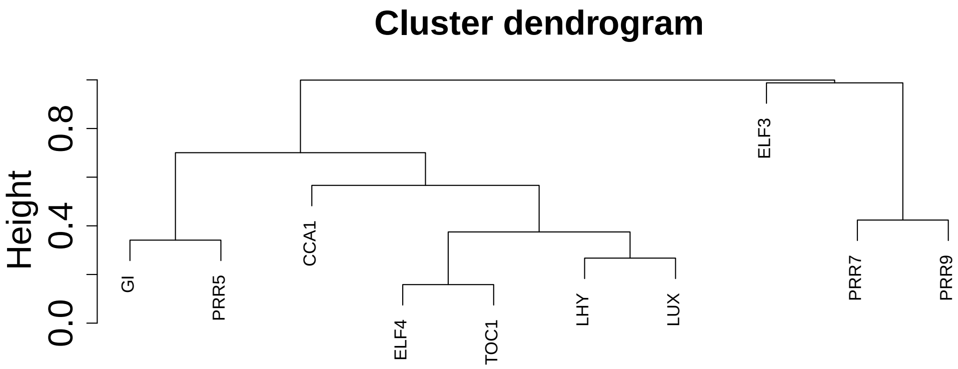

(b) Cluster dendrogram for the regulators

Figure S8: Correlations between regulators in the *Arabidopsis* circadian clock data set, Example 3. (a) Histogram of absolute correlations of regulators; self correlations (=1) are excluded. (b) Cluster dendrogram for the regulators. The regulators are grouped using a hierarchical clustering method (R package “hclust”), where the distance between every two regulators is measured by one minus their absolute correlation and the agglomeration method is “complete”.

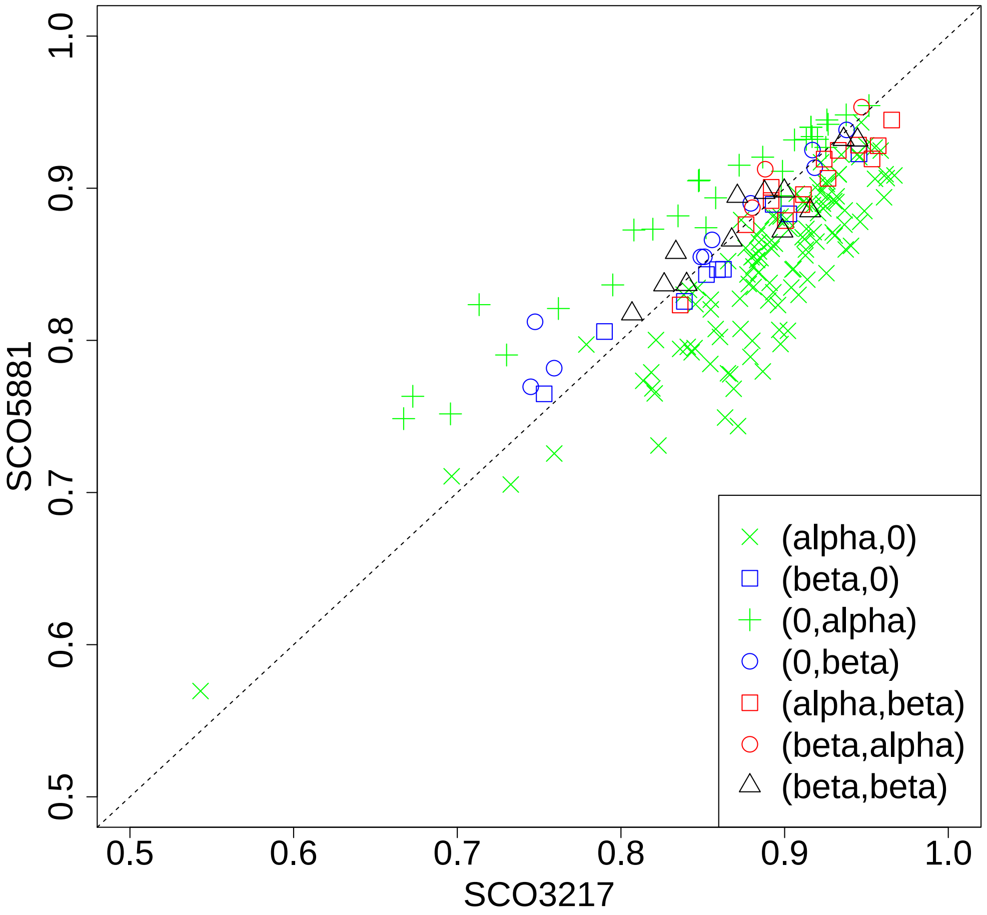

Figure S9: The absolute shifted correlations of SCO3217 and SCO5881 in the phosphate depletion data (Example 1) with selected targets categorised as follows: sole α targets of SCO3217 (green,$\times$), sole β targets of SCO3217 (blue, $\square$), sole α targets of SCO5881 (green,+), sole β targets of SCO5881 (blue,$\circ$), mixed α/β targets of SCO3217 and SCO5881(red,$\square$), mixed β/α targets of SCO3217 and SCO5881(red,$\circ$), and complete β targets of SCO3217 and SCO5881 (black,Δ). The absolute shifted correlation is the absolute value of the correlation between the expression data of the regulator at time points 26:43 hrs and that of the target at 27:44 hrs, accounting for the 1 hr causal shift in equation (1) of main text. Target classification corresponds to the ICN N(0.95,0.55).

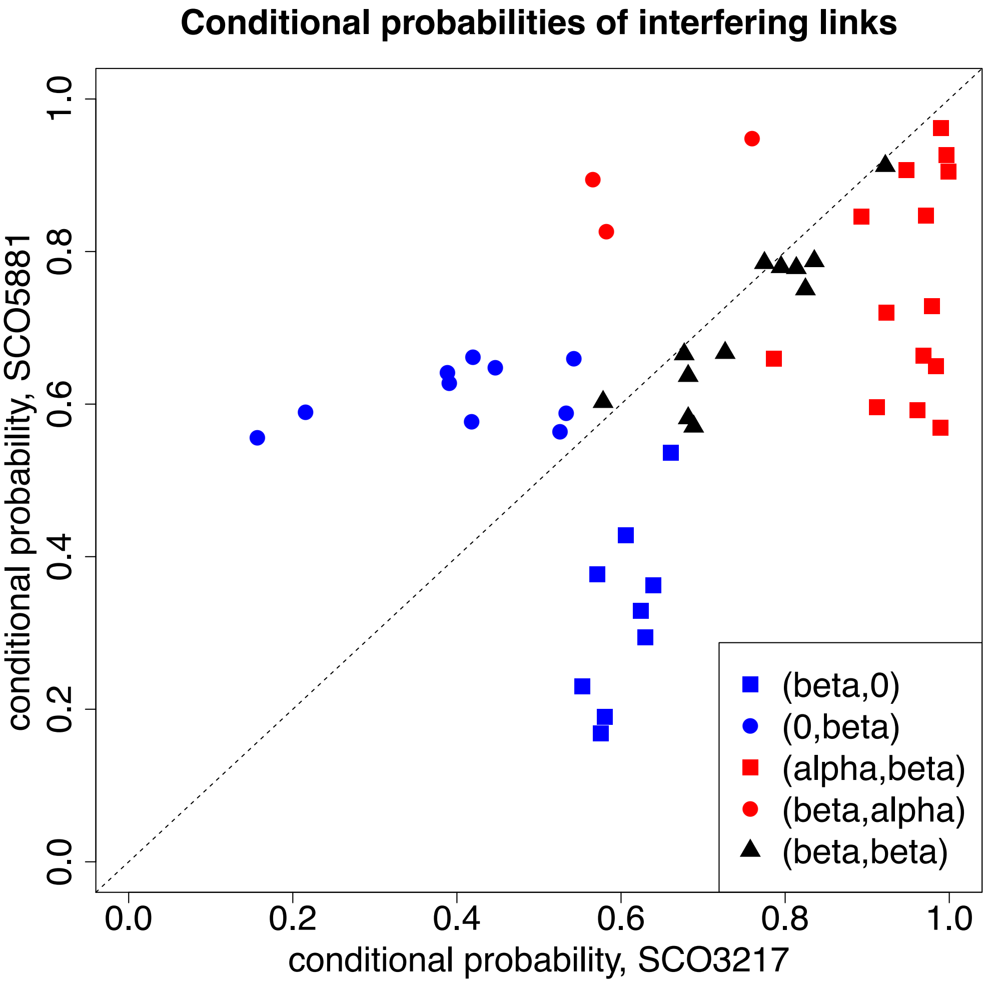

Figure S10: Scatter plot of conditional probabilities of the interfering links between SCO3217 and SCO5881 in the ICN N(0.95,0.55) for the phosphate depletion data set. The conditional probabilities for pairs of links containing at least one β-link are plotted: sole β-links of SCO3217 (blue, filled $\square$), sole β-links of SCO5881 (blue, filled $\circ$), mixed α/β-links of SCO3217 and SCO5881 (red, filled $\square$), mixed β/α-links of SCO3217 and SCO5881(red, filled $\circ$), and complete β-links of SCO3217 and SCO5881 (black, filled Δ).

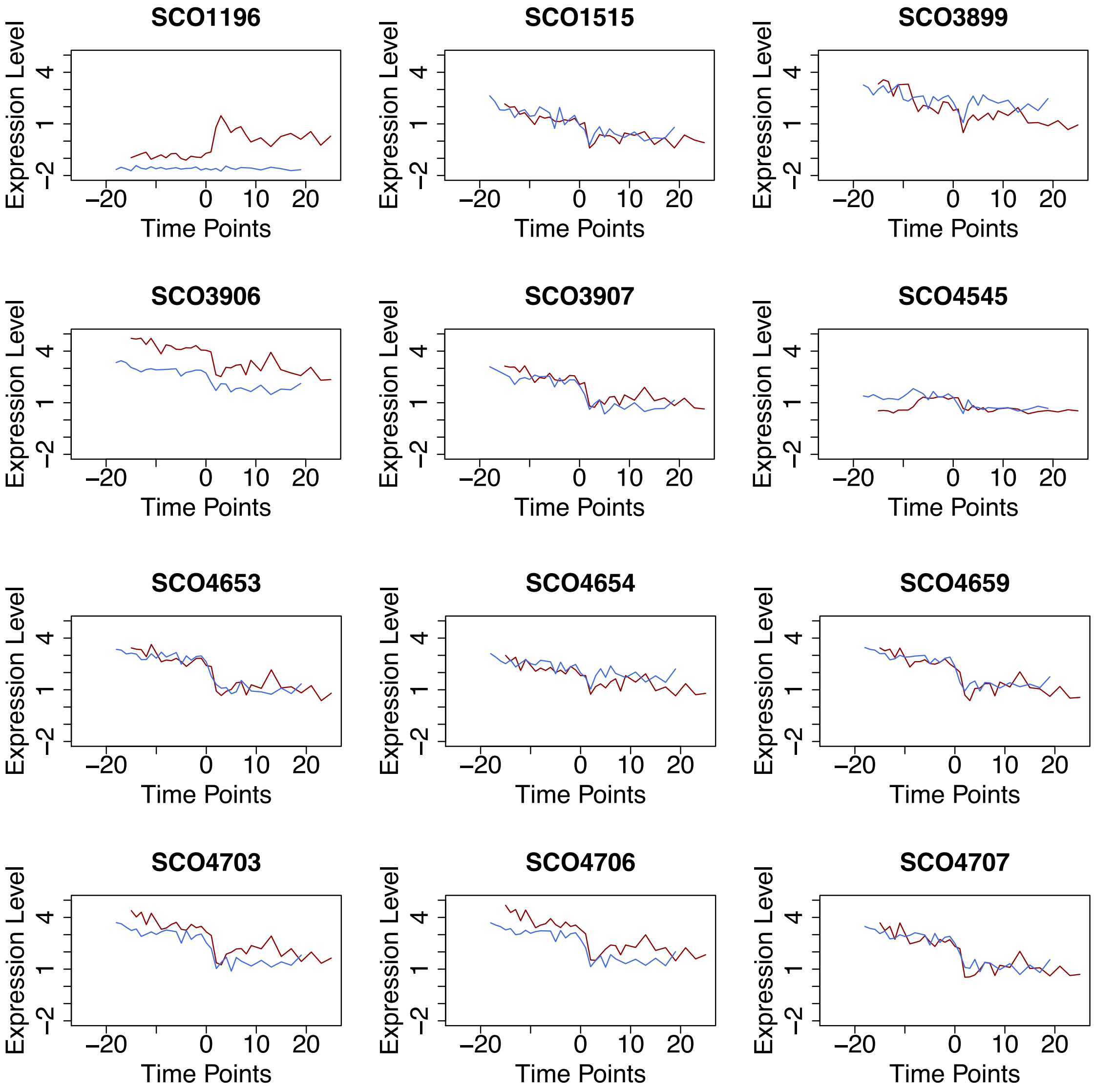

Figure S11: Plots of PhoP targets for both the wild type under phosphate depletion data (red) and the ΔphoP mutant data (blue). In each plot, the 0 hour refers to 35 hours for the wild type and 41 hours for the mutant when phosphate is depleted in the respective fermentor. All time points in the WT data set are plotted, section 1.1, although network inference on the WT is restricted to 26-44 hrs. Data is normalised in each data set to have mean 0, variance 1 across time and over all genes in the array.

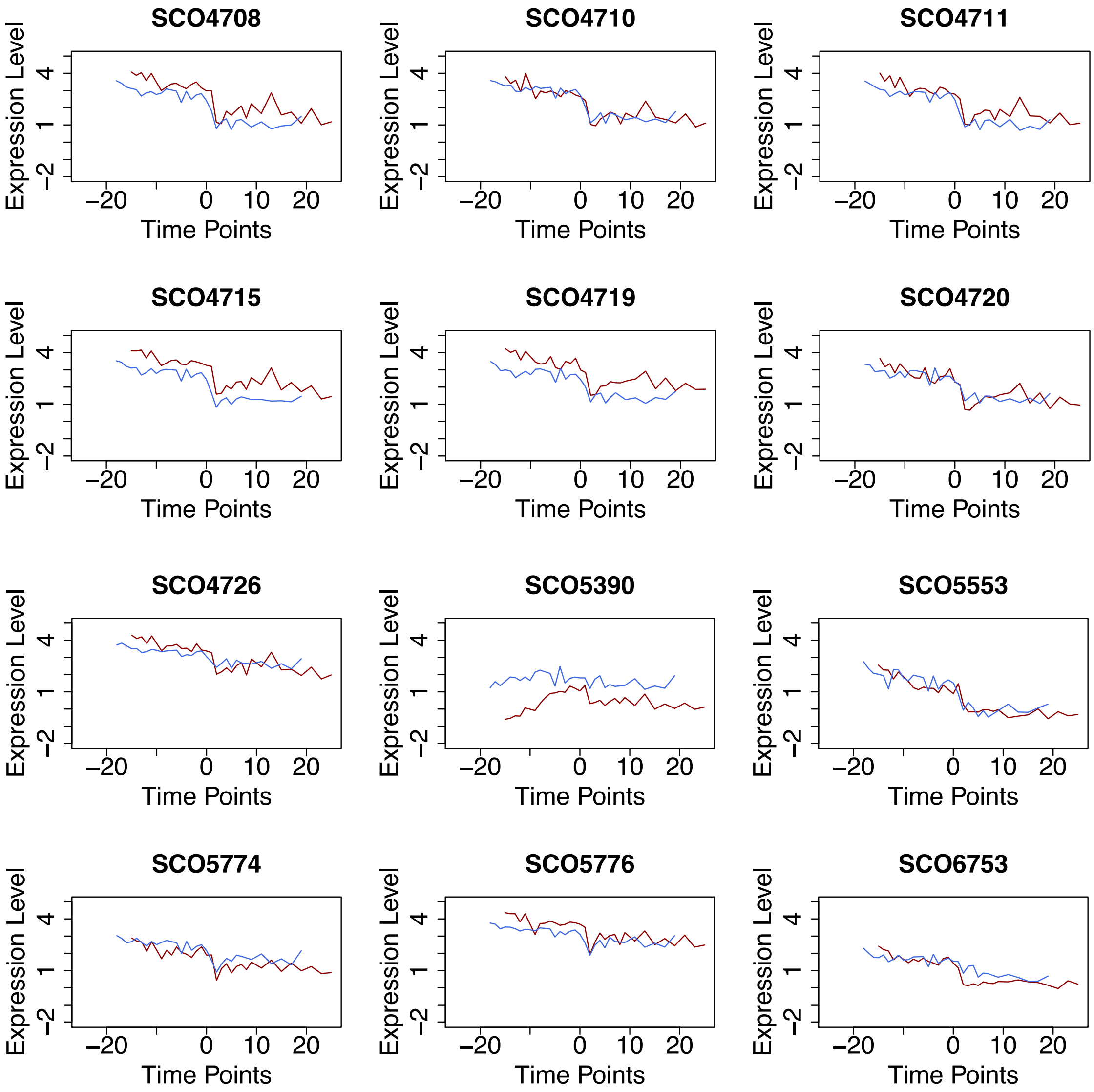

Figure S11: Continued.

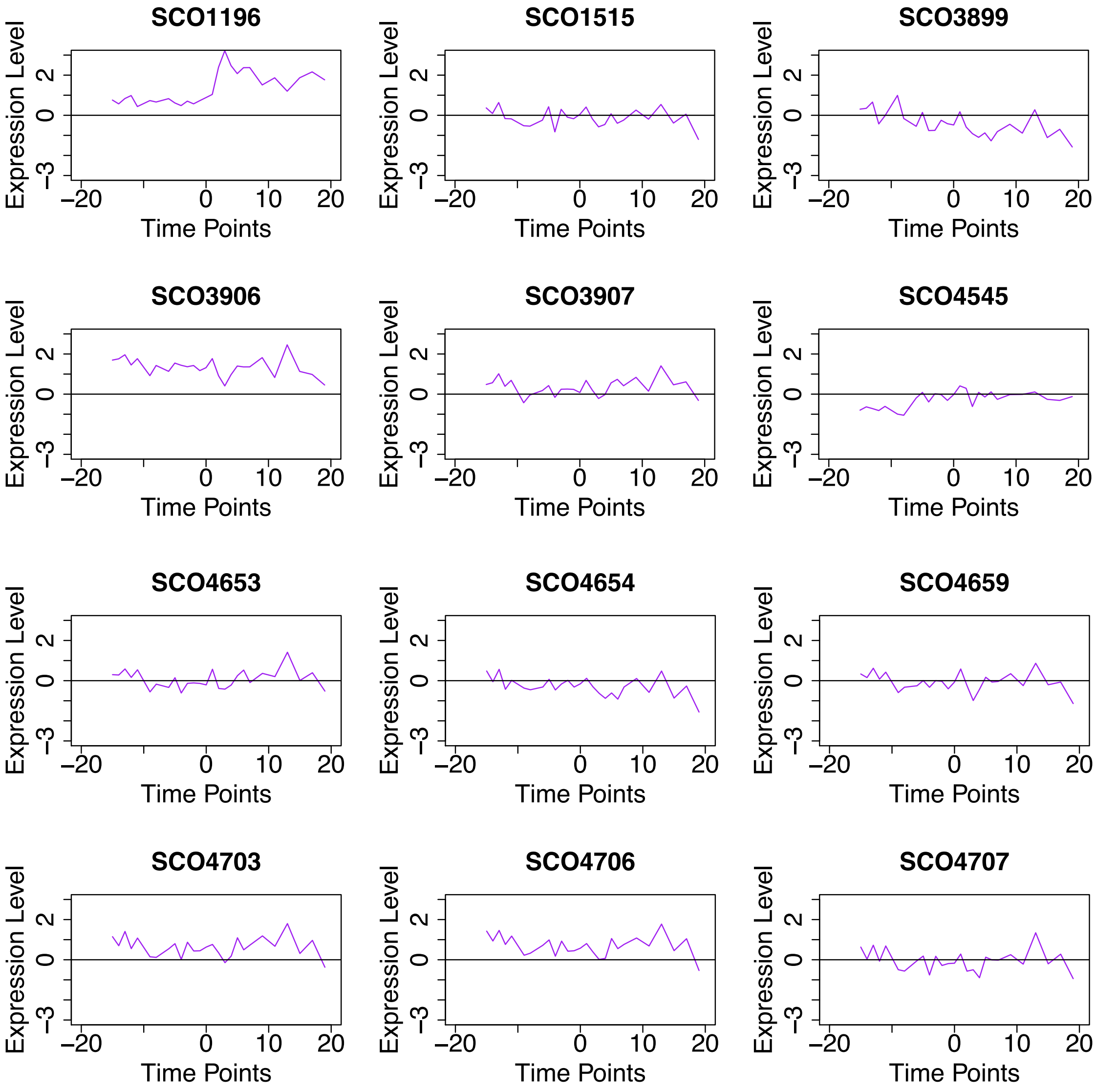

Figure S12: Plots of the gene expression difference between $\Delta phoP$ mutant and the wild type under phosphate depletion for genes in Figure S11. In each plot, the 0 hour refers to 35 hours for the wild type and 41 hours for the mutant when phosphate is depleted in the respective experiment. Common time points where difference is shown are at -15, -14, -13, -12, -11, -9, -8, -6, -5, -4, -3, -2, -1, 0, 1, 2, 3, 4, 5, 6, 7, 9, 11, 13, 15, 17, 19. They are 20 hrs-24 hrs at 1 hr resolution, 26-27 hrs at 1 hr resolution, 29-42 hrs at 1 hr resolution, and 44-54 hrs at 2 hrs resolution for wild type data; 26-30 hrs at 1 hr resolution, 32-33 hrs at 1 hr resolution, 35-48 hrs at 1 hr resolution, and 50-60 hrs at 2 hrs resolution for $\Delta phoP$ data.

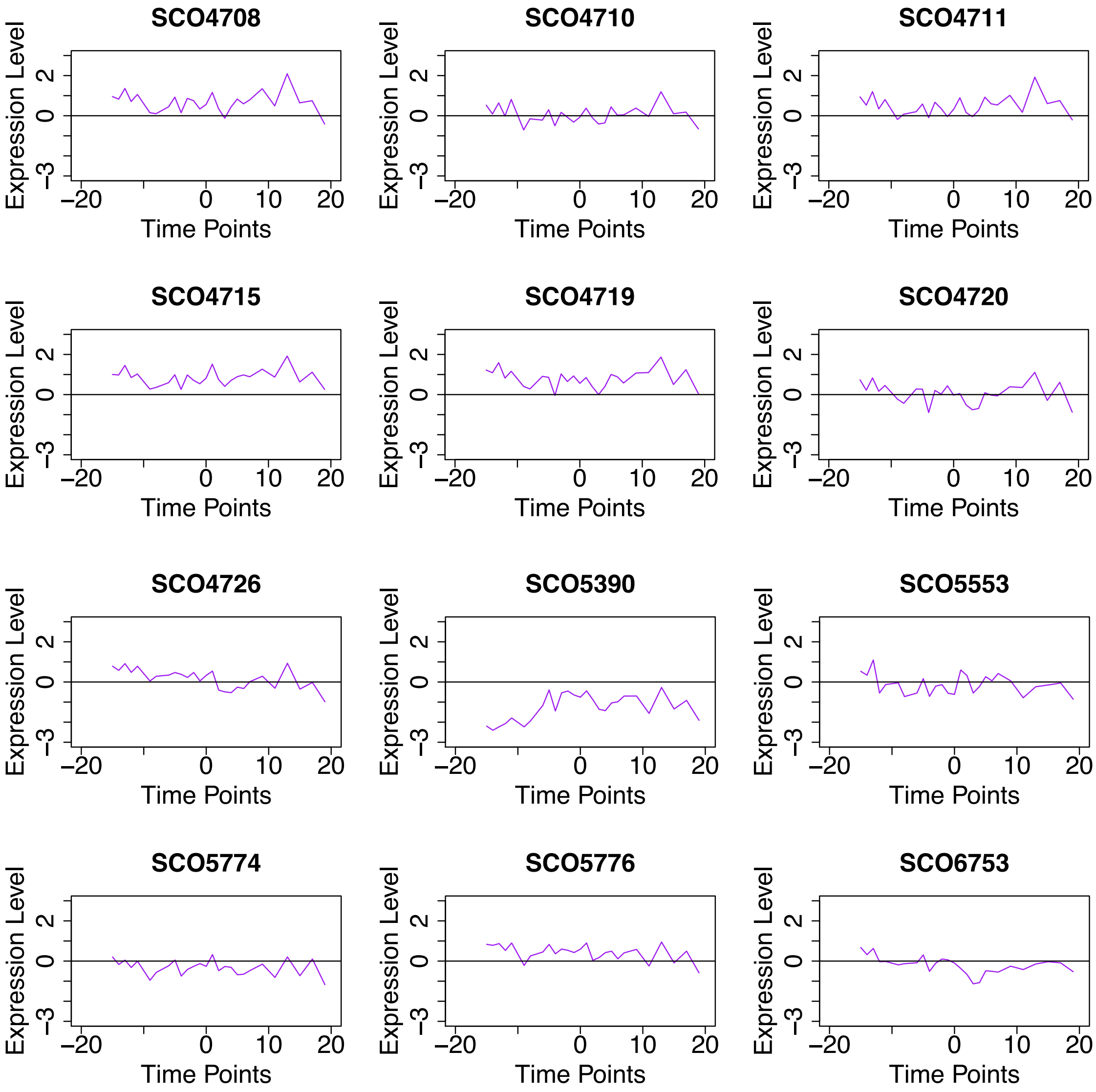

Figure S12: Continued.

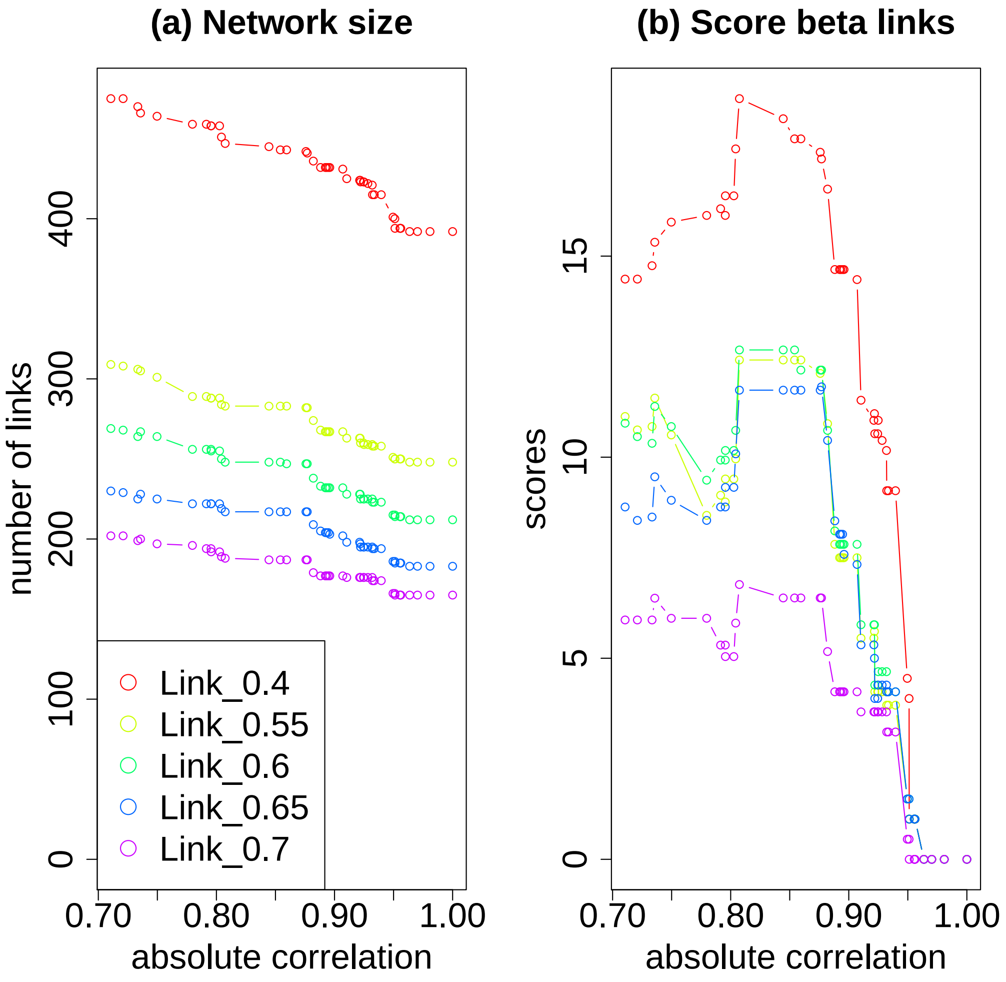

Figure S13: Analysis of the β-link composition of the *S. coelicolor* glutamate depletion ICN with respect to link ϕ and correlation ω thresholds. The total number of links (a) and the score of β-links (b) for networks $N(\omega, \phi)$ are plotted against the absolute correlation threshold ω between regulators for link thresholds ϕ =0.4, 0.55, 0.6, 0.65, 0.7, see legend. At the correlation threshold $\omega = 1$, the network comprises α-links only determined from the posterior link probabilities.

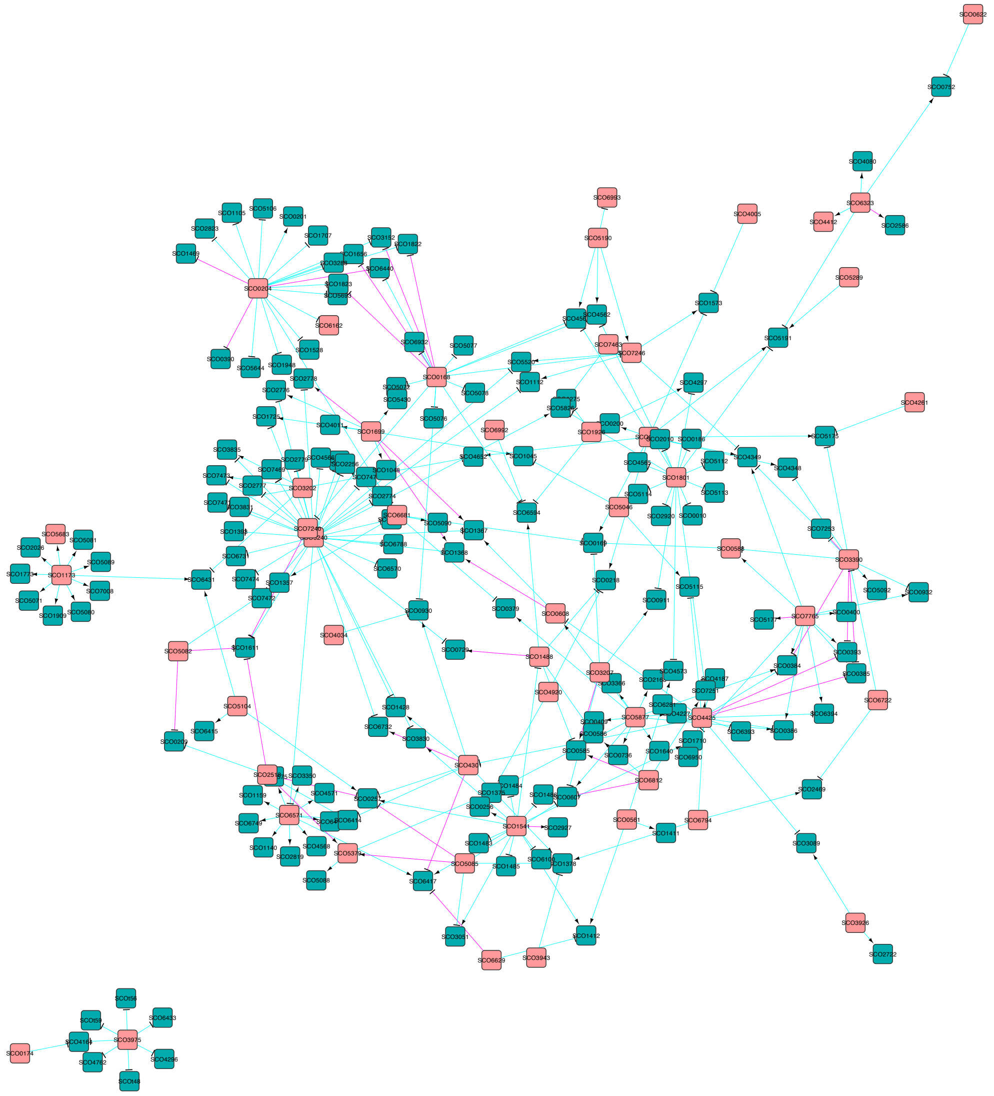

Figure S14: Cytoscape visualisation of the glutamate depletion ICN $N(\omega, \phi)$ with link threshold 0.55 and (optimal) correlation threshold 0.859. Nodes are regulators (pink), and non-regulator target genes (blue). T-arrow and arrow head links refer to inhibited and activated regulation. There are 248 α-links and 35 β-links.

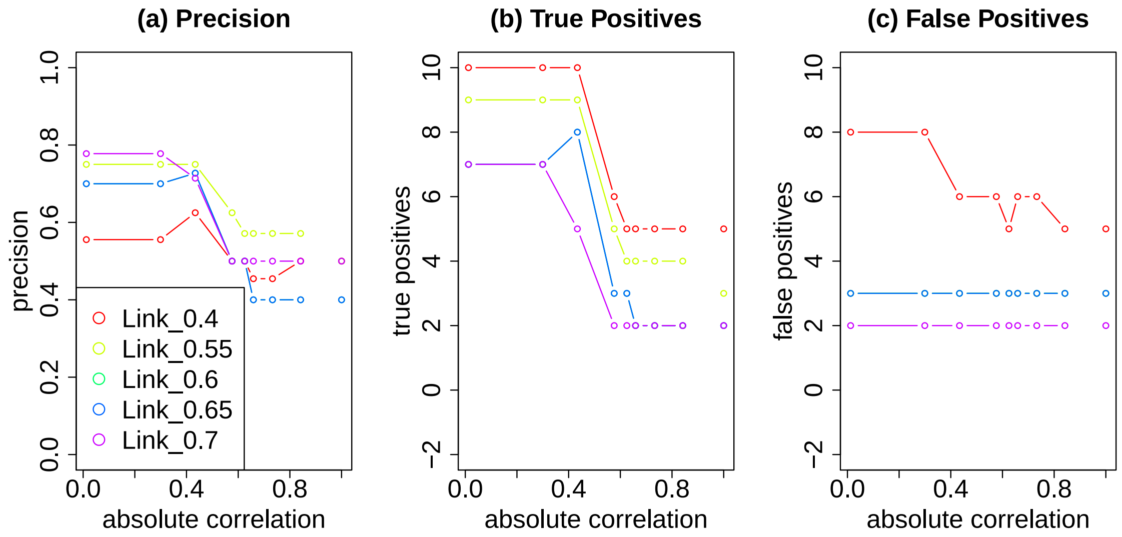

Figure S15: Evaluation of predicted links in the ICN with respect to link ϕ and correlation ω thresholds for the *Arabidopsis* circadian clock data. The precision (a), the true positives, TP, (b) and the false positives, FP, (c) for networks $N(\omega, \phi)$ are plotted against the absolute correlation threshold ω between regulators for link thresholds ϕ =0.4, 0.55, 0.6, 0.65, 0.7, see legend. At the correlation threshold $\omega = 1$, the network comprises α-links only determined from the posterior link probabilities, giving the precison and TP, FP of the uncorrected network. The ground truth network is defined by the *Arabidopsis* circadian clock model in (Pokhilko et al., 2013), Table S4. A link in an ICN is considered to be a true positive if it has the same direction and regulation type as in the ground truth network, otherwise, it is a false positive. The precision is computed as TP/(TP+FP).

6 Supplementary Tables

Table S3: Validation of PhoP targets. The respective columns are: predicted PhoP targets, the absolute correlation in gene expression between the target and PhoP, the Bayes factor (BF) computed from BATS (Angelini et al., 2008) on the difference in the gene expression between wild type strain and the ΔphoP mutant under phosphate depletion, PHO box word score and PHO box dyad score. Targets marked ** (*) have a dramatic change in their dynamics with BF less than 0.005 (0.05).

Target	AbsCor	BF	WordScore	DyadScore
SCO1196**	0.62	3.77×10^{-8}	NA	NA
SCO1515	0.60	1.39×10^{-1}	4.14	NA
SCO3899**	0.52	8.66×10^{-4}	12.14	8.10
SCO3906	0.59	1.72×10^{-1}	2.47	NA
SCO3907	0.59	3.23×10^{-1}	2.75	NA
SCO4545*	0.35	1.01×10^{-2}	11.49	7.73
SCO4653	0.60	1.01×10^{-1}	11.77	NA
SCO4654*	0.59	1.06×10^{-2}	4.14	3.26
SCO4659	0.65	1.19×10^{-1}	3.21	4.19
SCO4703	0.68	1.23×10^{-1}	10.47	8.47
SCO4706*	0.68	1.15×10^{-2}	10.47	8.47
SCO4707	0.64	1.07×10^{-1}	10.47	8.47
SCO4708	0.60	1.06×10^{-1}	10.47	8.47
SCO4710	0.64	3.01×10^{-1}	10.47	8.47
SCO4711	0.65	2.11×10^{-1}	10.47	8.47
SCO4715	0.60	6.56×10^{-1}	10.47	8.47
SCO4719	0.71	1.40×10^{-1}	2.56	4.75
SCO4720	0.69	6.81×10^{-2}	2.56	4.75
SCO4726**	0.58	1.41×10^{-3}	12.89	7.73
SCO5390**	0.10	2.62×10^{-6}	NA	NA
SCO5553	0.42	1.78×10^{-1}	NA	NA
SCO5774	0.57	3.54×10^{-1}	4.24	NA
SCO5776	0.58	1.85×10^{-1}	4.24	NA
SCO6753**	0.64	1.39×10^{-3}	3.96	6.98

Table S4: The benchmark *Arabidopsis* circadian clock network. 0 denotes absence of a link, 1 denotes an activating link, and -1 an inhibitory link. Links are derived from the *Arabidopsis* circadian clock model in (Pokhilko et al., 2013). Since we do not have data on the Evening Complex (EC) expression, we include in the model the three genes comprising the EC seperately, *i.e.* LUX, ELF4 and ELF3; variation in the transcription of any of these genes could affect the concentration of the EC complex.

Target/Regulator	LHY	CCA1	PRR9	PRR7	PRR5	TOC1	GI	LUX	ELF4	ELF3
LHY	0	0	-1	-1	-1	-1	0	0	0	0
CCA1	0	0	-1	-1	-1	-1	0	0	0	0
PRR9	1	1	0	0	0	-1	0	-1	-1	-1
PRR7	1	1	1	0	0	-1	0	0	0	0
PRR5	1	1	0	1	0	-1	0	0	0	0
TOC1	-1	-1	0	0	0	0	0	-1	-1	-1
GI	-1	-1	0	0	0	-1	0	-1	-1	-1
LUX	-1	-1	0	0	0	-1	0	-1	-1	-1
ELF4	-1	-1	0	0	0	-1	0	-1	-1	-1
ELF3	-1	-1	0	0	0	0	0	0	0	0

Table S5: Comparison between NIACS and Bayes Net Toolbox (BNT) on *Arabidopsis* circadian clock data. The BNT algorithm (learn_struct_dbn_reveal.m, https://code.google.com/p/bnt) needs discrete valued time series data. Thus, at each time point in a time course a gene is assigned the value 1 if its expression level is lower than its average level across all time points in the time series, otherwise it is assigned the value 2. The input data for BNT can be either multiple time series (4 biological replicates) or single time series (median of the biological replicates). Bayesian Information Criterion (BIC) is used in BNT to determine the network. The inferred networks are compared with the ground truth network in Table S4, calculating the true positives, false positives and precision. Since BNT can not predict the regulation type (activation or inhibition), a link in the network inferred by BNT is counted as a true positive (TP) if it gives the right causal relationship, otherwise, it is a false positive (FP). In contrast a link in the network inferred by NIACS is considered to be a true positive (TP) if it has the same direction and regulation type as in the ground truth network, otherwise, it is a false positive (FP). The precision is computed as TP/(TP+FP).

Algorithm	Data Type	True Positives	False Positives	Precision
NIACS	Replicates, Continuous	9	3	0.75
BNT	Replicates, Discrete	8	11	0.42
BNT	Median, Discrete	7	9	0.44

Table S6: Causal signal synergy between regulators in the three optimal ICNs. For each network the following are tabulated: the clusters containing synergistic regulators, the minimum of their absolute correlations (Min. Abs. Corr), their common target, their link type (0 denotes non-link, α denotes an α-link), the posterior link probabilities (PP), the posterior conditional probabilities (CP) and the absolute correlations between each regulator and its target, $r_{reg-target}$.

	Phosphate depletion ICN(0.865,0.550)		
Regulators	(SCO4228,4229)	(SCO1488,6265)	(SCO3323,3943,5819)
Min Abs. Corr.	0.968	0.915	0.868
Target	SCO3220	SCO4636	SCO1254
Link type	(α,α)	(α,α)	(0,α,α)
PP	(0.673,0.668)	(0.925,0.910)	(0.002,0.679,0.650)
CP	(0.021,0.006)	(0.183,0.015)	(0.003,0.089,0.008)
$r_{reg-target}$	(0.628,0.570)	(0.784,0.873)	(0.586,0.618,0.707)
	Glutamate depletion ICN(0.859,0.550)		
Regulators	(SCO4034,4301,6629)	(SCO3202,5104)	(SCO2954,6681,7246,7765)
Min Abs. Corr.	0.924	0.895	0.876
Target	SCO0930	SCO6431	SCO4349
Link type	(α,α,0)	(α,α)	(0,0,α,α)
PP	(0.969,0.970,0.003)	(0.761,0.696)	(0.039,0.008,0.606,0.624)
CP	(0.003,0.004,0.002)	(0.216,0.004)	(0.019,0.017,0.027,0.073)
$r_{reg-target}$	(0.868,0.870,0.898)	(0.264,0.025)	(0.088,0.046,0.099,0.194)
	Arabidopsis circadian clock ICN(0.434,0.550)		
Regulators		(ELF4,LHY,LUX,TOC1)	
Min Abs. Corr.		0.625	
Target		PRR5	
Link type		(α,α,0,0)	
PP		(0.756,0.815,0.086,0.100)	
CP		(0.079,0.204,0.040,0.149)	
$r_{reg-target}$		(0.475,0.604,0.625,0.460)	

7 List of cytoscape files

- PhosphateDepletionCytoICN.cys: the cytoscape file for the *S. coelicolor* phosphate depletion ICN(0.865, 0.550).

- GlutamateDepletionCytoICN.cys: the cytoscape file for the *S. coelicolor* glutamate depletion ICN(0.859, 0.550).

- CircadianClockCytoICN.cys: the cytoscape file for the *Arabidopsis* circadian clock ICN(0.434,0.550).

References

Bansal,M. et al. (2006) Inference of gene regulatory networks and compound mode of action from time course data. *Bioinformatics*, 22, 815-822.

Angelini,C. et al. (2008) BATS: a Bayesian user-friendly software for analyzing time series microarray experiments. *BMC Bioinformatics*, 9, 415.

Breeze,E. et al. (2011) High-resolution temporal profiling of transcripts during *Arabidopsis* leaf senescence reveals a distinct chronology of processes and regulation. *Plant Cell*, 23, 873-894.

Morrissey,E.R. et al. (2010) On reverse engineering of gene interaction networks using time course data with repeated measurements. *Bioinformatics*, 26, 2305-2312.

Nieselt,K. et al. (2010) The dynamic architecture of the metabolic switch in *Streptomyces coelicolor*. *BMC Genomics*, 11, 10.

Penfold,C.A. and Wild, D.L. (2011) How to infer gene networks from expression profiles, revisited. *Interface Focus*, 1, 857–870.

Pokhilko,A. et al. (2013) Modelling the widespread effects of TOC1 signalling on the plant circadian clock and its outputs. *BMC Syst. Biol.*, 7, 23.

Thomas,L. et al. (2012) Metabolic switches and adaptations deduced from the proteomes of *Streptomyces coelicolor* wild type and phoP mutant grown in batch culture. *Mol. Cell Proteomics*, 11, M111.013797.

Waldvogel,E. et al. (2011) The P_{II} protein GlnK is a pleiotropic regulator for morphological differentiation and secondary metabolism in *Streptomyces coelicolor*. *Appl. Microbiol. Biotechnol.*, 92, 1219-1236.

Wentzel,A. et al. (2012) Optimized submerged batch fermentation strategy for systems scale studies of metabolic switching in *Streptomyces coelicolor* A3(2). *BMC Syst. Biol.*, 6, 59.

Windram,O. et al. (2012) *Arabidopsis* defence against *Botrytis cinerea*: chronology and regulation deciphered by high-resolution temporal transcriptomic analysis. *Plant Cell*, 24, 3530-3557.

Wu,H. et al. (2003) MAANOVA: A software package for the analysis of spotted cDNA microarray experiments. In G. Parmigiani, E. Garrett, R. Irizarry, and S. Zeger (Eds.), *The Analysis of Gene Expression Data: Methods and Software* (pp. 313-341). New York: Springer.
